# Are Hotspots Always Hotspots? The Relationship between Diversity, Resource and Ecosystem Functions in the Arctic

**DOI:** 10.1371/journal.pone.0074077

**Published:** 2013-09-10

**Authors:** Heike Link, Dieter Piepenburg, Philippe Archambault

**Affiliations:** 1 Institut des sciences de la mer de Rimouski, Université du Québec à Rimouski, Rimouski, Québec, Canada; 2 Mainz Academy of Sciences, the Humanities and Literature, Institute for Polar Ecology of the University of Kiel, Kiel, Germany; University of Connecticut, United States of America

## Abstract

The diversity-ecosystem function relationship is an important topic in ecology but has not received much attention in Arctic environments, and has rarely been tested for its stability in time. We studied the temporal variability of benthic ecosystem functioning at hotspots (sites with high benthic boundary fluxes) and coldspots (sites with lower fluxes) across two years in the Canadian Arctic. Benthic remineralisation function was measured as fluxes of oxygen, silicic acid, phosphate, nitrate and nitrite at the sediment-water interface. In addition we determined sediment pigment concentration and taxonomic and functional macrobenthic diversity. To separate temporal from spatial variability, we sampled the same nine sites from the Mackenzie Shelf to Baffin Bay during the same season (summer or fall) in 2008 and 2009. We observed that temporal variability of benthic remineralisation function at hotspots is higher than at coldspots and that taxonomic and functional macrobenthic diversity did not change significantly between years. Temporal variability of food availability (i.e., sediment surface pigment concentration) seemed higher at coldspot than at hotspot areas. Sediment chlorophyll a (Chl *a*) concentration, taxonomic richness, total abundance, water depth and abundance of the largest gallery-burrowing polychaete *Lumbrineristetraura* together explained 42% of the total variation in fluxes. Food supply proxies (i.e., sediment Chl *a* and depth) split hot- from coldspot stations and explained variation on the axis of temporal variability, and macrofaunal community parameters explained variation mostly along the axis separating eastern from western sites with hot- or coldspot regimes. We conclude that variability in benthic remineralisation function, food supply and diversity will react to climate change on different time scales, and that their interactive effects may hide the detection of progressive change, particularly at hotspots. Time-series of benthic functions and its related parameters should be conducted at both hot- and coldspots to produce reliable predictive models.

## Introduction

A question that is central in ecology is: can diversity of communities predict ecosystem functions? This question has been feeding the experimental efforts and discussions of terrestrial and marine ecologists for some time [1,2]. However, very few studies have explicitly investigated the relationship between biodiversity and ecosystem functions at higher latitudes [3]. Ecosystem functioning, defined as the biogeochemical and biotic processes and interactions in an ecosystem, is strongly related to ecosystem services providing, e.g., wood or fish for human needs [4]. With global changes underway, such ecosystem services are threatened and, hence, efforts are increasing to define the role of biodiversity and its changes for ecosystem functions [5]. Particularly, hotspots of species richness are considered an insurance of functioning in the face of species loss [6]. Other studies have demonstrated the importance of resource availability modifying the diversity- ecosystem function relationship [7–10].

Polar ecosystems are of particular interest, because climate changes are affecting them faster and stronger than the ecosystems in other regions [11]. There is thus a need for particularly rapid assessment of how environmental changes may alter ecosystem functioning in polar latitudes. In contrast to most other oceans, more than half of the Arctic Ocean’s total area consists of rather shallow continental shelves. Understanding shelf-environments is indispensable for a description of the marine Arctic ecosystems and their functioning. While recent reviews have achieved an inventory of benthic diversity of Canadian and pan-Arctic shelves [12,13], their benthic ecosystem functioning is understudied [14]. In soft-bottom environments, which dominate Arctic continental shelves, the degradation of organic matter and coupled oxygen and inorganic nutrient fluxes from the sediments back to water column is an important ecosystem function [15,16]. Benthic oxygen consumption is generally linked to the availability of food resources, which are often measured as sediment pigments [17–19], but other nutrient fluxes are less often integrated in ecological analyses. Knowledge on the remineralisation of other nutrients from the sediments in the Canadian Arctic has only recently been reported [20], and is not directly correlated to oxygen fluxes [21]; [22]. This underlines the need to investigate which factors influence benthic remineralisation function as a whole. In other habitats and experimental studies for example, the role of the number and identity of species for oxygen consumption and nutrient fluxes at the sediment-water interface have been clearly demonstrated [23–25]. Moreover, the functional diversity seems to be more important than number of taxa [26].

At sites with higher benthic remineralisation than known on average from the Canadian Arctic [20,27], which we define as **hotspots**, we have recently found high temporal variability of oxygen consumption. However, sites characterized by a generally low oxygen consumption (hereafter ‘**coldspots**’) showed lower temporal variability [28–30]. Time-series data from deep-sea sites also report important interannual variability of the downward export of organic matter [31], but changes in macrofaunal benthic community composition are observed only after several years or decades [32]. While temporal and regional variabilities in benthic oxygen uptake in the Arctic have so far been linked to environmental factors and food supply, it is not clear to which extent the benthic diversity affects benthic oxygen uptake or even multiple benthic fluxes [3,33].

For most areas of the Arctic, predictive models have to rely on few benthic data collected from different locations at different times, because Arctic time-series studies are even rarer than in other oceans [34,35]. But the use of such datasets to detect directional (progressive) change can be affected by the natural variability (stochastic change) of marine systems [34]. Climate forced environmental changes introduce additional temporal and spatial variability of ecosystem functioning. Here we assess how diversity (taxonomic and functional; composition, species number and abundance) and environmental factors affect spatial *vs* temporal variability of ecosystem functions at hotspots and coldspots in the Canadian Arctic. The objectives of this study were to distinguish between the temporal and spatial variation in a multivariate benthic ecosystem function in the Canadian Arctic, and to investigate the relation of the often used function proxies diversity and food supply with spatio-temporal variation. The resampling of study sites in the same season of different years for multiple remineralisation fluxes, diversity and sediment pigments was the key approach to allow for separating temporal from spatial variation in the diversity-ecosystem function analysis.

We test specifically the following hypotheses: (1) Benthic remineralisation function is significantly different between years at hotspots but not at coldspots, (2) food availability for the benthos (measured as sediment pigments) is significantly different between years at hotspots but not at coldspots, (3) Taxonomic community composition is not significantly different between years, (4) Functional community composition is not significantly different between years, and (5) Food supply explains temporal variation and macrofaunal community parameters (e.g., total abundance, richness) explain spatial variation in benthic remineralisation functioning. The results will allow evaluating whether diversity can serve as a reliable surrogate for benthic remineralisation function in the Canadian Arctic despite temporal variability in polar ecosystem processes.

## Methods

### Study region

**Figure 1:**
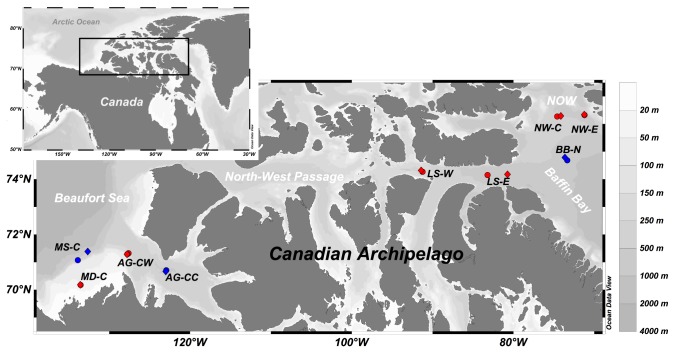
Locations of sites sampled across the Canadian Arctic in 2008 and 2009. Red = hotspots, blue = coldspots; circle = 2008, diamonds = 2009. Note that one point on the map can represent two sampling events when exact relocation was achieved.

The study covered the benthic ecosystems in shelf environments across the Canadian Arctic Archipelago from the eastern Mackenzie Shelf in the west to the North Water Polynya (NOW) in Northern Baffin Bay in the east (Figure 1). These environments are characterised by strong seasonality, with the productive period being subjected to the timing of ice-melt and increasing light duration as summer arrives.

The eastern Beaufort Sea and Amundsen Gulf are dominated by coastal shelves down to 600 m water depth. Pelagic primary production ranges from 30 to 70 g C m^-2^ yr^-1^, indicating generally oligotrophic conditions [36]. Rather low primary production was also found in summer and fall 2005-2007 in the eastern Beaufort Sea, with daily production rates of 73 ± 37 mg C m^−2^ d^−1^ [37]. In the Cape Bathurst Polynya at the eastern boundary of the Amundsen Gulf, however, annual production rates are higher, reaching 90 to 175 g C m^-2^ yr^-1^ [38]. Ardyna et al. [37] reported daily primary production rates in summer of 159 ± 123 mg C m^−2^ d^−1^, and intensive phytoplankton blooms related to ice-edge upwelling events were documented for coastal regions of the Mackenzie Shelf and Amundsen Gulf in 2008 [30,39]. Annual vertical POC fluxes of 1.6–1.8 g C m^-2^ yr^-1^ and 2.4 g C m^-2^ yr^-1^ were estimated at 200 m water depth for the Mackenzie Shelf and the Cape Bathurst Polynya, respectively [40–42]. Seafloor sediments are usually composed of more than 70% silt and clay [43].

The eastern North-West-Passage is marked by the opening of Lancaster Sound into Baffin Bay. From its western limitation at the Barrow Strait sill (125 m) the channel reaches a depth of more than 800 m in the sound itself. Tidal and bathymetry induced mixing of Pacific and Atlantic waters east of Barrow Strait allow for high primary production with rates of 251 ± 203 mg C m^−2^ d^−1^ [37] and an annual mean of about 60 g C m^-2^ yr^-1^ [44,45]. Vertical export can be high [46] and studies on benthic biomass and diversity report values that are among the highest known from the Arctic [47], but important gaps of data need to be filled for a more comprehensive description of the area [13,44,48].

The North Water Polynya (NOW) is located in Baffin Bay north of Lancaster Sound. It opens each year depending on latent heat fluxes, ice-bridge formation and northerly winds over the Nares Strait between western Greenland and eastern Ellesmere Island [49,50]. Its productivity is considered to be the highest in the Arctic with primary production reaching values up to 250 g C m^-2^ yr^-1^ in the east [51] and 150 g C m^-2^ yr^-1^ across the region [36,37]. A significant amount of organic carbon is exported to water depths of > 200 m with highest values in the western polynya [52]. The seabed under the polynya reaches a depth of around 700 m and studies on benthic carbon turnover showed comparatively low rates in 1998 [53] and higher rates in 2008 [20]. Abundance of benthic fauna was highest in the NOW center, indicating the role of currents and possible advection processes in the food supply for the benthos [54].

### Field sampling

Samples presented here were collected from nine sites (MD-C, AG-CW, LS-W, LS-E, NW–C, NW-E (hotspots) and MS-C, AG-CC, BB–N (coldspots), Figure 1) distributed across the study region in 2008 and 2009. To avoid confounding influence of seasons, the same sites were sampled in the same season each year. Sampling was conducted onboard the CCGS Amundsen between July and October during the Circumpolar Flaw Lead Study [55], ArcticNet expeditions in collaboration with the Canadian Healthy Ocean Network [56] and the Malina project (http://malina.obs-vlfr.fr/). Locations were chosen to study both hotspots and coldspots in the Canadian Arctic. Prior to sampling, a hotspot site was defined from previous knowledge (in order of importance, if available) as a site in an area known for high benthic oxygen fluxes, primary productivity, and/or vertical export (Mackenzie Delta plume, Cape Bathurst Polynya, Lancaster Sound, NOW). A coldspot site was defined as the opposite, outside such areas, which generally show low benthic fluxes. More information on the definition, categorisation and verification of benthic hotspots is found in Darnis et al. [20] as well as Kenchington et al. [27]. At each sampling event (‘station’), an USNEL box corer was deployed for seafloor sediment collection. From each box core, three to five sub-cores of ten cm diameter and approximately 20 cm sediment depth were taken for assessing benthic remineralisation function (i.e., benthic oxygen demand and nutrient remineralisation) in shipboard microcosm incubations (Table 1). After incubation, the same sediment cores were passed through a 0.5 mm mesh sieve under slow running seawater. The sieve residues were preserved in a 4% seawater-formaldehyde solution for later analyses of species diversity and abundance under a dissection microscope. Sediment surface (first cm) of additional three sub-cores were stored in pre-weighed plastic vials and frozen immediately at -80 °C to determine food supply (i.e., sedimentary Chl *a* and phaeopigment concentrations) later in the laboratory (Table 1). Part of the flux and sediment pigment data presented here has been published as part of studies with a more oceanographic focus (Table 1 [20,22,29,30]). Sampling licenses were obtained for the North-West Territories (Canada) by the Aurora Research Institute (#14304R, #14543), by the Environmental Impact Screening Committee (#03 09 03), and by the Department of Fisheries and Oceans (DFO) (# S-09/10-4013-IN) and for Nunavut (Canada) by the Nunavut Research Institute (#0501408R-M, #0504609R-M) and by DFO (# S-08/09-1043-NU, # S-09/10-1049-NU).

**Table 1:** Station list with labels, date of sampling, geographic position, number of within-station replicate samples used to determine each benthic boundary flux and diversity (BBF) and food supply proxies (sedimentary Chl a, and phaeopigments), and references (Ref), if data has been published.

**Regime**	**Site**	**Station label**	**Date**	**Depth [m]**	**Latitude [°N]**	**Longitude [°W]**	**BBF (n)**	**Food proxy (n)**	**Ref**
Hotspot	MD-C	I-434	30/Jun/08	45	70.177	133.537	4	3	20,30
		M-390	31/Jul/09	47	70.178	133.569	3	3	22
Hotspot	AG-CW	I-408	25/Jul/08	206	71.323	127.606	3	3	20
		M-140	07/Aug/09	154	71.285	127.783	3	3	20
Hotspot	LS-W	A-304	06/Sep/08	353	74.271	91.248	3	3	20
		A-304	23/Oct/09	331	74.318	91.406	3	3	
Hotspot	LS-E	A-301	08/Sep/08	707	74.153	83.209	3	3	20
		A-323	25/Oct/09	786	74.172	80.726	3	3	
Hotspot	NW–C	A-108	14/Sep/08	444	76.270	74.594	3	3	20
		A-109	28/Oct/09	451	76.290	74.137	3	3	
Hotspot	NW-E	A-115	13/Sep/08	668	76.326	71.215	3	3	20
		A-115	29/Oct/09	669	76.335	71.238	3	3	
Coldspot	MS-C	I-435	02/Jul/08	318	71.072	133.876	4	3	20,30
		M-345	16/Aug/09	577	71.382	132.652	3	3	22
Coldspot	AG-CC	I-405	21/Jul/08	596	70.707	122.939	5	3	20,30,29
		A-405	16/Oct/09	559	70.665	122.996	3	3	22
Coldspot	BB–N	A-136	10/Sep/08	795	74.786	73.633	3	3	20
		A-136	30/Oct/09	810	74.687	73.349	3	3	

For sub-regions: AG = Amundsen Gulf, MD = Mackenzie Delta, MS = Mackenzie Shelf/Slope; LS = Lancaster Sound; NW = North Water Polynya; = BB = Baffin Bay; C, E, N, W = central, east, north, west; A = ArcticNet expedition, I = IPY Circumpolar Flaw Lead Study, M = Malina Project. For References [20]: = oxygen fluxes, silicic acid fluxes, phosphate fluxes and food proxy [22]; = oxygen fluxes, silicic acid fluxes, phosphate fluxes and food proxy [29]; = oxygen fluxes, food proxy and benthic biomass [30]; = oxygen fluxes and food proxy.

### Benthic remineralisation function

Benthic remineralisation function was measured as the multivariate combination of benthic oxygen, nitrate, silicic acid, phosphate and nitrite fluxes at the sediment-water interface (benthic boundary fluxes). For this, shipboard incubations of sediment microcosms were run in a dark, temperature-controlled room (2 to 4 °C) for 24 to 48 h. Total sediment oxygen flux was determined as the decrease in oxygen concentrations in the water phase and was measured periodically (2 to 8 h intervals) with a non-invasive optical probe (Fibox 3 LCD, PreSens, Regensburg, Germany). To determine changes in nutrient concentrations, samples of the overlying water phase were taken at three times during the incubation, including the onset and end.

Oxygen and nutrient fluxes were determined as the slope of the linear regression of the oxygen and nutrient concentration on incubation time and corrected for solute concentration in the replacement water. A more detailed description of this method can be found in Link et al. [22,29].

### Food supply

Food supply was measured as Chl *a* and phaeopigment concentrations. They were analysed fluorometrically following a modified protocol proposed by Riaux-Gobin and Klein [57] as described in Link et al. [29]. Two grams of wet substrate were incubated with 10 ml 90% Acetone (v/v) for 24 h at 4 °C, and the supernatant was measured in a Turner Design 20 fluorometer before and after acidification. Chl *a* and total pigment concentration (Chl *a* + phaeopigments) were determined. Quantities are expressed as microgram pigment per gram of dry sediment [µg g^-1^].

### Macrofaunal diversity

#### Taxonomic diversity

Sediment residues from the sieved incubation cores were sorted under a dissection microscope in the lab to retrieve benthic organisms that were subsequently identified to the lowest possible taxonomic level and counted (abundance, N). Taxa not identified to the species level were distinguished from other specimens (e.g. sp. 1) and classified as morpho-species. Where such consistency across the study region was not achieved (e.g., due to a lack of describable characters), specimens were grouped into the lowest common taxon (e.g., *Sipuncula*). Taxonomic richness is the number of taxa at each station (Tax S or S_Tax_).

#### Functional diversity

Consequently, species were classified into functional groups according to their traits in terms of feeding mode, body size, mobility and bioturbation influence (Table 2, Table S1) [58–60]. Categories were chosen based on their presumed influence on benthic remineralisation. Species were allowed more than one trait for feeding mode. Trait information was retrieved from the best resources available [61–68]. For analyses of composition and richness, functional groups were treated in the same way as taxonomic entities. Functional group richness is the number of different categories of traits per station (S_Func_).

**Table 2:** Functional traits.

**Category**	**Level**
Feeding/Diet	C = Carnivorous (predator or passive suspension)
	D = Surface deposit feeder
	F = Filter/Suspension feeder
	O = Omnivorous (scavenger)
	P = Parasite
	S = Subsurface deposit feeder
Size	S < 3 mm
	3 mm < M < 10 mm
	L > 10 mm
Mobility	M = Mobile
	S = Sessile
	H = Hemimobile
Bioturbation	B = Active burrower (diffusive)
	G = Gallery burrower
	S = Surface dweller
	T = Tube burrower

Categories of traits and their levels used to define functional groups for taxa.

### Statistical analyses

We used a mixed-model PERMANOVA design to test for temporal and spatial differences in (a) remineralisation function (b) food supply proxies (i.e., sediment pigment concentrations), (c) taxonomic and (d) functional composition. The factors ‘year’ (two levels: 2008, 2009), fully crossed with ‘regime’ (two levels: hotspot, coldspot), ‘sites’ nested in ‘regime’ (six hotspots: MD-C, AG-CW, LS-W, LS-E, NW–C, NW-E, and three coldspots: MS-C, AG-CC, BB–N) and their interactions were tested. The resemblance matrices quantifying the between-replicate similarities in terms of all five standardized fluxes (O_2_ and four nutrients) and the two sediment pigments were calculated based on Euclidean distances. Missing data points were replaced using the ‘missing’ function in PRIMER-E software [69]. Taxonomic and functional abundance matrices were fourth-root transformed and their resemblance matrices were calculated based on Bray-Curtis similarity [69]. PERMANOVA pair-wise tests were run for significant sources of variation between the factors [70]. The significance level was corrected for multiple testing using the Bonferoni correction with α_B_ = α/n, where n is the number of comparisons and α=0.05. Homogeneity of dispersion could not be tested for groups of the interaction terms 'year x site (regime)' using the PERMDISP routine due to the small sample size (n = 3) [70]. Instead, we determined average squared distances (Euclidian, for fluxes and pigments) or dissimilarities (Bray-Curtis, for taxonomic and functional composition) across sites within and between years for samples of hotspots and coldspots, respectively, using the SIMPER routine [69]. Multidimensional Scaling (MDS) plots were used to visualize the resemblance patterns.

A stepwise distance-based linear model permutation test (DistLM [71]) was performed to identify which subset of biotic and environmental variables best predicted the multivariate variation of five benthic boundary fluxes at 18 stations (all stations of 2008-2009). Ten predicting variables were entered into the model: sediment surface Chl *a* concentration, sediment surface phaeopigment concentration, S_Tax_, S_Func_, N, Shannon-Wiener index, abundance of the largest gallery burrower *Lumbrineristetraura* (single species of its functional group) and abundance of the largest dominant tube-burrower group DFLHT, water depth and the date of ice-free conditions. Ice-free conditions were determined from weekly ice charts for the western and eastern Canadian Arctic published by the Canadian Ice Service (CIS) available on http://www.ec.gc.ca/glaces-ice/. A site was considered to be ice-free if ice concentrations below 1 prevailed for more than two consecutive weeks. To meet the linearity assumption for predictor variables, Chl *a* was ln transformed prior to analysis. No pair of variables was linearly correlated by r > 0.85 and hence all variables were retained for possible inclusion in the model. The stepwise routine was run employing 9999 permutations and using the AIC_c_ selection criterion. The AIC_c_ was devised to handle situations where the number of samples (*N*) is small relative to the number (v) of predictor variables (*N*/*v*<40) [70]. Results were visualized with a distance-based redundancy analysis (dbRDA) [70].

Data will be made available in 2014 as: Link H, Archambault P*, Piepenburg D (2014) Studying the functioning of benthic 'hotspot' vs 'coldspot' ecosystems in the Canadian Arctic metadata and data - A Canadian Healthy Oceans Network Marine Biodiversity Project, MB-01. Canadian Healthy Oceans Data Repository, Version 1. Available through the Canadian Healthy Oceans Network, Memorial University, St. John's, NF, Canada at http://chone.marinebiodiversity.ca/research.

## Results

### Temporal and spatial variability of benthic remineralisation function

**Figure 2:**
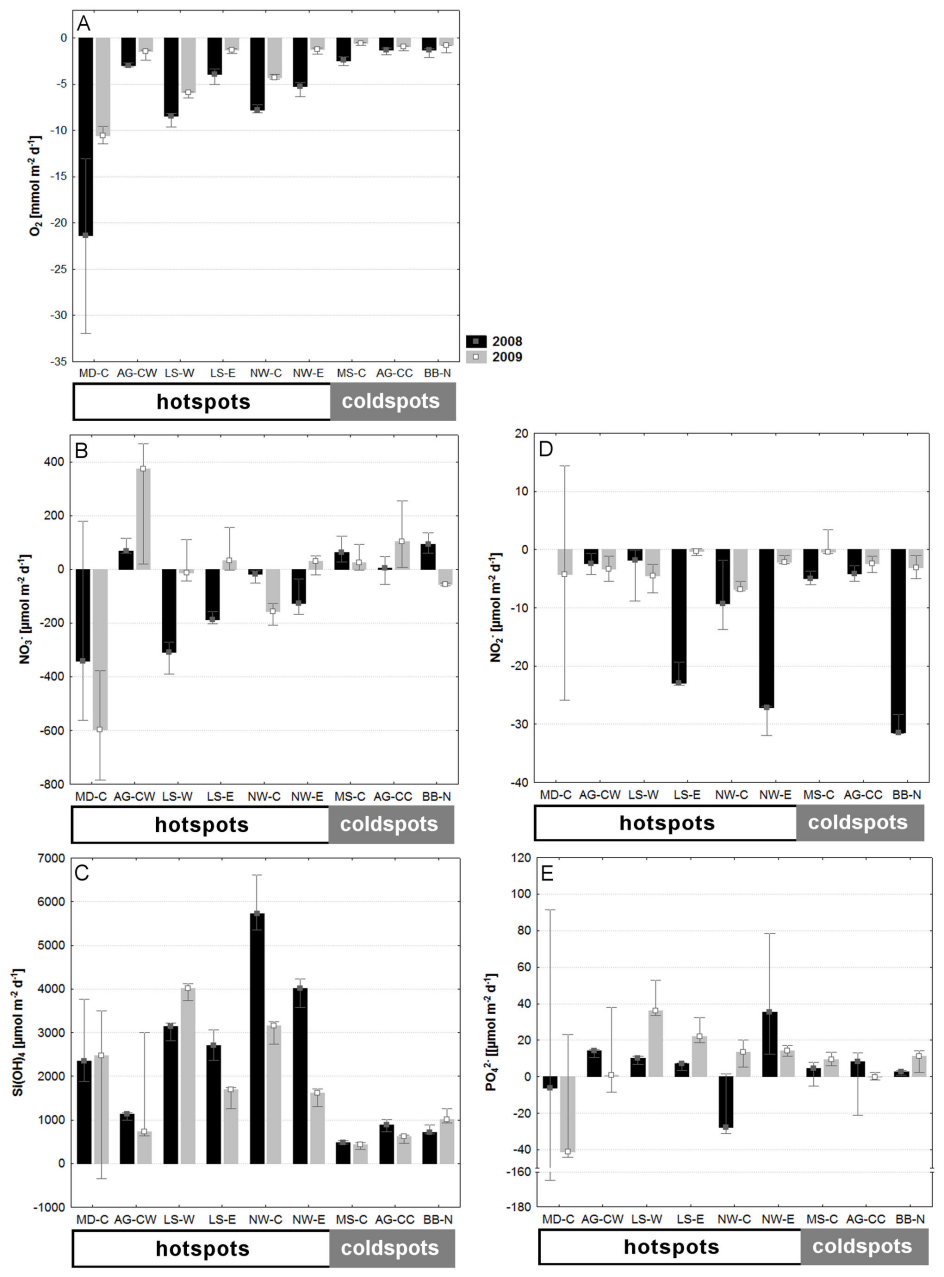
Benthic boundary fluxes at each sampling event across the Canadian Arctic in 2008 and 2009. A: Oxygen fluxes. B: Nitrate fluxes. C: Silicic Acid fluxes. D: Nitrite fluxes. E: Phosphate fluxes. Columns represent median ± min/max. For years: black columns = 2008, grey columns = 2009. For sub-regions: AG = Amundsen Gulf, MD = Mackenzie Delta, MS = Mackenzie Shelf/Slope; LS = Lancaster Sound; NW = North Water Polynya; = BB = Baffin Bay; C, E, N, W = central, east, north, west.

In general, benthic boundary fluxes were higher at hotspots than at coldspots and higher in 2008 than in 2009 (Figure 2). This pattern was most pronounced for oxygen fluxes, whereas other nutrient fluxes showed more heterogeneous patterns. Sites of greatest benthic oxygen and nitrate uptakes, and silicic acid and phosphate releases were MD-C, NW–C and LS-W (all hotspots) (Figure 2).

**Figure 3:**
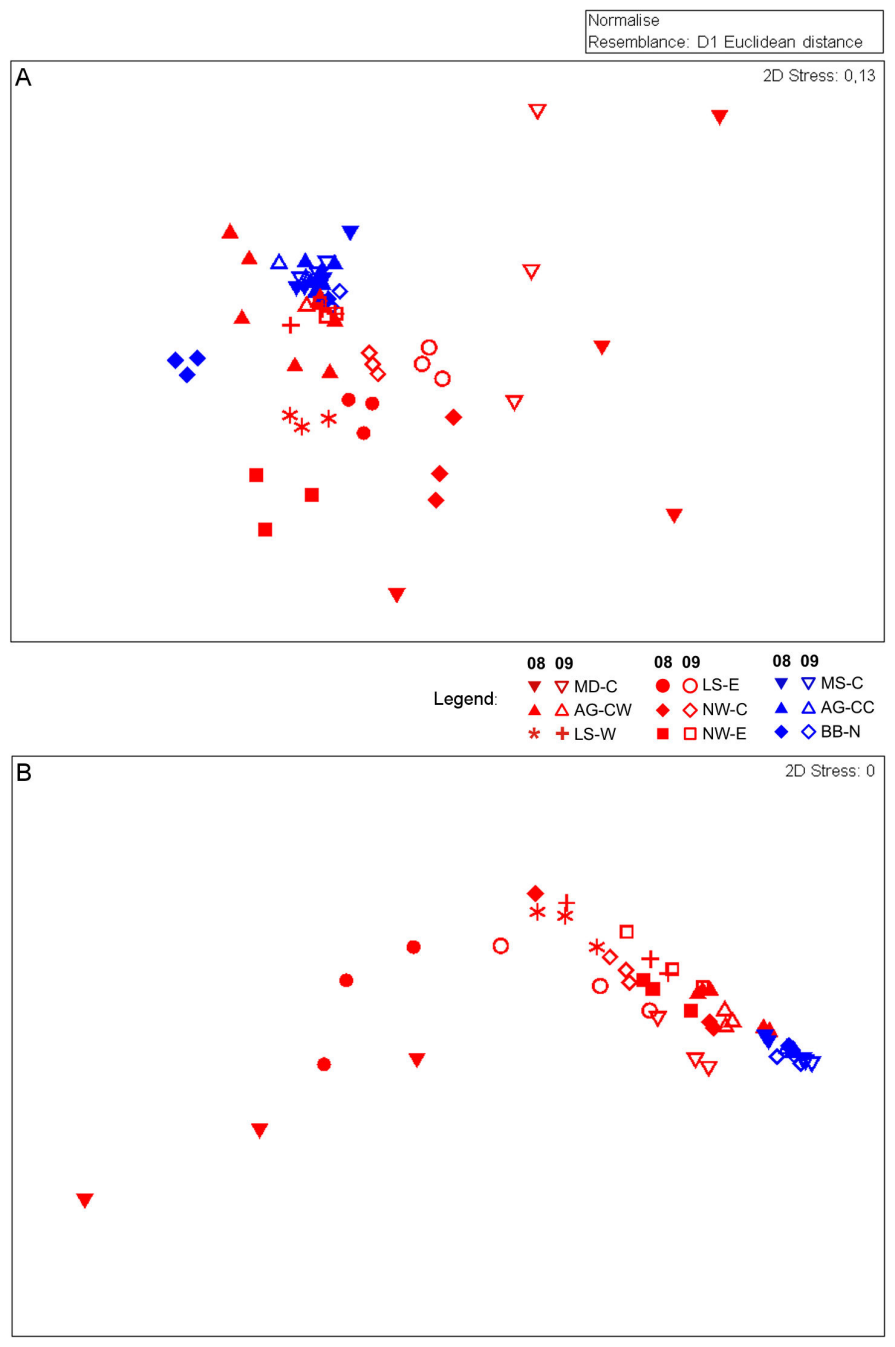
Temporal and spatial patterns of (A) benthic remineralisation function and (B) food supply at each sampling event across the Canadian Arctic in 2008 and 2009. The plot shows the relative distance of samples determined as Euclidian distance of (A) the five benthic boundary fluxes and (B) Chl *a* and phaeopigments. Red = hotspots, blue = coldspots; full symbols = 2008, open symbols = 2009; AG = Amundsen Gulf, MD = Mackenzie Delta, MS = Mackenzie Shelf/Slope; LS = Lancaster Sound; NW = North Water Polynya; = BB = Baffin Bay; C, E, N, W = central, east, north, west.

The multivariate composition of benthic boundary fluxes was significantly different between hotspots and coldspots and between sites, with a significant interaction between years and the nested factor sites (Table 3). The years 2008 and 2009 were significantly different at the sites LS-W, LS-E, NW–C, NW-E and BB–N (Table 4, Figure 3A). Variability within and between years was greater at hotspots than at coldspots (Table 5).

**Table 3:** Effects of factors on multivariate benthic parameters.

**Benthic Parameter**	**Source of variation**	**df**	**MS**	**Pseudo-F**	**P (perm)**
Benthic Boundary Fluxes	Year	1	17.099	2.219	0.1047
	Regime	1	43.911	3.031	0.0310
	Site(R)	7	14.066	6.977	0.0001
	Year x R	1	3.448	0.455	0.7892
	Year x Site(R)	7	7.497	3.719	0.0001
	Res	40	2.016		
	Total	57			
Pigments	Year	1	4.327	1.643	0.2436
	Regime	1	36.117	8.316	0.0226
	Site(R)	7	4.343	14.202	0.0001
	Year x R	1	2.551	0.969	0.3643
	Year x Site(R)	7	2.633	8.611	0.0001
	Res	36	0.306		
	Total	53			
Functional Compositions	Year	1	2056.0	1.115	0.3656
	Regime	1	16579.0	3.105	0.0032
	Site(R)	7	5181.4	9.496	0.0001
	Year x R	1	1986.8	1.078	0.3887
	Year x Site(R)	7	1801.8	3.302	0.0001
	Res	40	545.6		
	Total	57			
Taxonomic Diversity	Year	1	3293.2	1.063	0.4143
	Regime	1	20697.0	2.096	0.0267
	Site(R)	7	9588.0	8.916	0.0001
	Year x R	1	2338.5	0.758	0.6628
	Year x Site(R)	7	3035.5	2.823	0.0001
	Res	40	1075.4		
	Total	57			

Results are from permutational multivariate analyses of variance (PERMANOVAs) testing the effect of Year (Ye), Regime (R), Site (Si) nested within Regime and their interactions. Calculation is based on Euclidian distance for benthic boundary fluxes and pigments, and on Bray-Curtis similarity of fourth-root transformed data of functional and taxonomic community composition. Significance level at P < 0.05.

**Table 4:** Difference between years for pairs of each site based on multivariate benthic parameters.

	**Regime**	**Site**		**t**	**Df**	**P (MC)**	**2008-2009**	**2008**	**2009**
**Fluxes**	Hotspots	MD-C		0.969	5	0.4221	4.86	5.33	4.09
		AG-CW		1.390	4	0.1984	1.26	0.35	1.62
		LS-W		4.184	4	**0.0043**	2.14	0.82	0.81
		LS-E		7.568	4	**0.0003**	2.79	0.52	0.67
		NW–C		3.934	4	**0.0034**	2.49	1.36	0.51
		NW-E		5.004	4	**0.0012**	3.63	1.61	0.37
	Coldspots	MS-C		1.946	5	0.0631	1.12	0.95	0.50
		AG-CC		1.486	6	0.1560	0.83	0.67	0.82
		BB–N		11.942	4	**0.0003**	2.99	0.41	0.44
**Pigments**	Hotspots	MD-C		4.122	4	0.0136	3.81	2.08	0.43
		AG-CW		5.686	4	**0.0020**	0.33	0.08	0.11
		LS-W		3.350	4	0.0146	2.01	0.95	0.94
		LS-E		1.534	4	0.1939	0.70	0.41	0.73
		NW–C		0.454	4	0.6822	0.95	1.30	0.19
		NW-E		0.910	4	0.4456	0.44	0.33	0.56
	Coldspots	MS-C		14.072	4	**0.0003**	0.42	0.04	0.05
		AG-CC		8.351	4	**0.0013**	0.28	0.05	0.05
		BB–N		1.328	4	0.2345	0.13	0.04	0.15
**F Comp**	Hotspots	MD-C	1.750		5	0.0493	56.51	61.29	69.03
		AG-CW	2.302		4	0.0199	39.89	62.98	60.85
		LS-W	1.791		4	0.0573	66.48	77.98	71.62
		LS-E	1.224		4	0.2451	66.86	67.41	71.21
		NW–C	2.691		4	0.0136	52.95	70.55	76.47
		NW-E	1.649		4	0.0774	67.30	75.61	72.13
	Coldspots	MS-C	2.223		5	0.0143	49.52	66.88	63.87
		AG-CC	1.167		6	0.2695	55.10	59.67	53.39
		BB–N	1.863		4	0.0549	66.74	72.50	78.15
**T Comp**	Hotspots	MD-C	1.579		5	0.0661	42.32	53.58	49.82
		AG-CW	2.098		4	0.0261	24.70	46.64	50.45
		LS-W	1.532		4	0.0988	47.29	58.87	53.80
		LS-E	1.602		4	0.0744	50.32	59.78	59.50
		NW–C	2.250		4	0.0230	34.64	61.77	53.98
		NW-E	1.316		4	0.1734	52.83	56.22	59.31
	Coldspots	MS-C	1.732		5	0.0389	35.99	46.58	52.79
		AG-CC	1.119		6	0.3012	45.23	47.42	45.95
		BB–N	1.796		4	0.0498	54.34	64.33	66.64

Results for PERMANOVAs pair-wise tests for the significant interaction term Year x Site (R) (Table 3) and the average distance (Fluxes, Pigments) or average similarity [%] (F Comp, T Comp) between (2008-2009) and within (2008, 2009) groups of replicates are presented. Calculation is based on Euclidian distance for benthic boundary fluxes and pigments and on Bray-Curtis similarity of fourth-root transformed data of functional and taxonomic community composition. Significance level after Bonferroni correction: P (MC) < 0.006. Significant results are in bold. For sub-regions: AG = Amundsen Gulf, MD = Mackenzie Delta, MS = Mackenzie Shelf/Slope; LS = Lancaster Sound; NW = North Water Polynya; = BB = Baffin Bay; C, E, N, W = central, east, north, west.

**Table 5:** Dispersion within and between 2008 and 2009 for hotspots and coldspots across the nested sites.

	**Group**	**Hotspots**	**Coldspots**
Fluxes	2008	5.08	0.34
(Sq. Eucl. Dist.)	2009	1.80	0.20
	*2008 vs 2009*	*10.66*	*3.08*
Pigments	2008	0.72	<0.01
(Sq. Eucl. Dist.)	2009	0.20	0.01
	*2008 vs 2009*	*3.73*	*0.09*
Tax. Comp	2008	44.22	50.17
(Dissimilarity)	2009	45.52	44.88
	*2008 vs 2009*	*57.97*	*55.57*
Func. Comp	2008	29.63	36.82
(Dissimilarity)	2009	31.07	34.86
	*2008 vs 2009*	*41.76*	*43.74*

Average squared distance (benthic boundary fluxes and pigments) and average squared Bray-Curtis dissimilarity calculated by SIMPER are presented.

### Temporal and spatial variability of food supply

Sediment Chl *a* concentrations ranged from 0.04 to 32.44 µg g^-1^ with highest values at the hotspot sites MD-C and LS-W and lowest values at coldspots. Sediment phaeopigment concentrations were higher at hotspots than at coldspots (Table S2).

Significant differences in the multivariate composition of sediment pigments were found between hotspots and coldspots and between sites, with a significant interaction between years and sites (Table 3). There were significant differences between 2008 and 2009 at sites AG-CW, MS-C and AG-CC (Table 4, Figure 3B). Variability within and between years was greater at hotspots than at coldspots (Table 5, Figure 3B).

### Temporal and spatial variability of taxonomic diversity

We identified a total of 311 macrofaunal taxa in the sediments taken from the incubation cores (Table S1). Taxonomic richness (S_Tax_) per core ranged from seven taxa at AG-CC (coldspot) up to 45 at LS-W (hotspot) (Table S2). Lowest abundance was found at sites MD-C and AG-CW (both hotspots) and highest in the NOW sites (hotspot) (Table S2).

**Figure 4:**
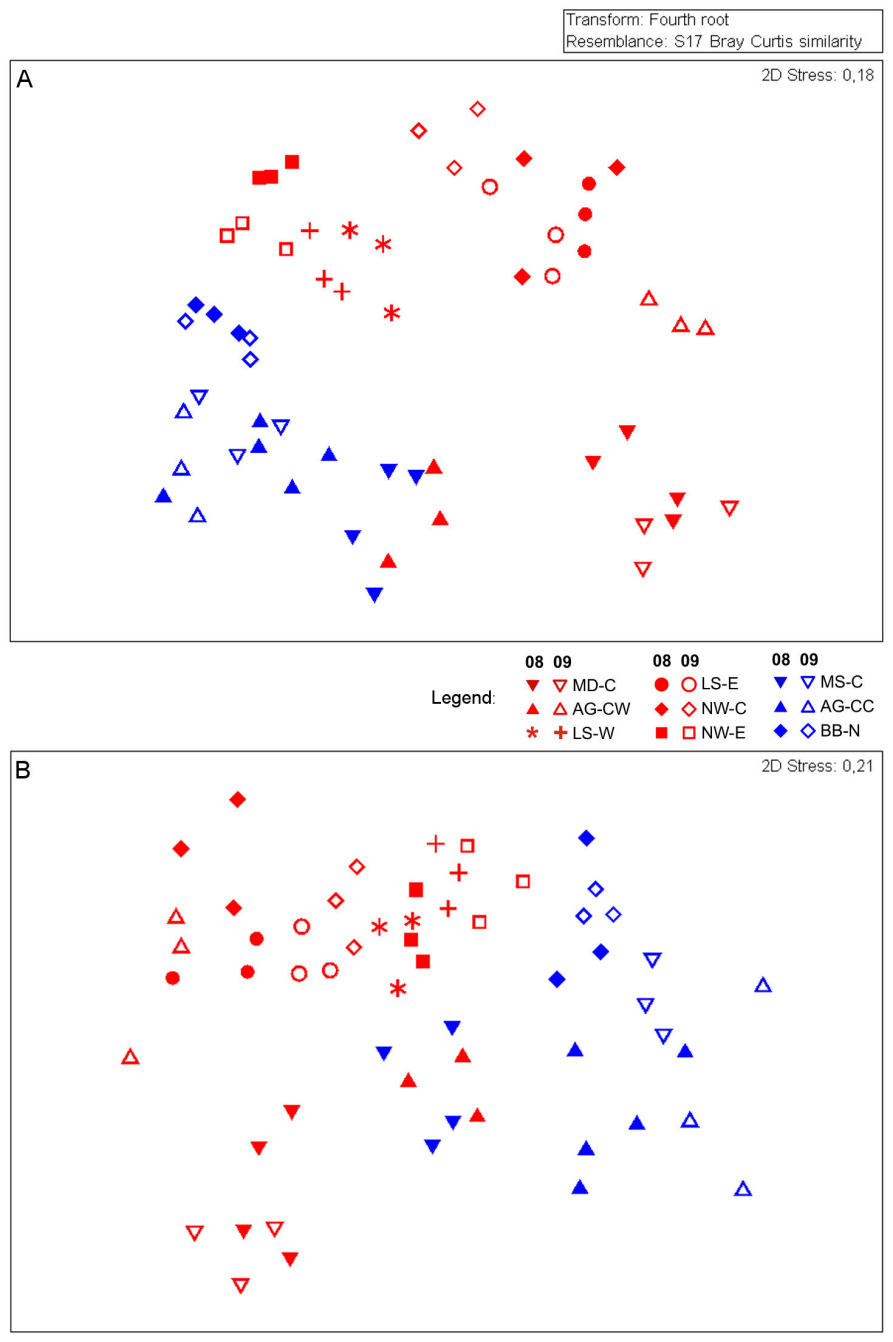
Temporal and spatial patterns of (A) taxonomic and (B) functional community composition at each sampling event across the Canadian Arctic in 2008 and 2009. The plot shows the relative similarity of samples in a multidimensional space determined as Bray-Curtis similarity based on 4^th^-root transformed abundance of (A) taxa and (B) functional groups. Red = hotspots, blue = coldspots; full symbols = 2008, open symbols = 2009; AG = Amundsen Gulf, MD = Mackenzie Delta, MS = Mackenzie Shelf/Slope; LS = Lancaster Sound; NW = North Water Polynya; = BB = Baffin Bay; C, E, N, W = central, east, north, west.

Taxonomic composition of communities was significantly different between hotspots and coldspots and between sites, with a significant interaction between years and sites (Table 3, Figure 4A). The consecutive years 2008 and 2009 were not significantly different at any site (Table 4). Intra-annual and inter-annual dissimilarities of hotspot and coldspot communities were comparable (Table 5, Figure 4A).

### Temporal and spatial variability of functional diversity

Taxa were classified into a total of 72 functional groups (Table S1). Number of functional groups (S_Func_) per core ranged between six groups at AG-CC (coldspot) and 32 at LS-W (hotspot) (Table S2).

Functional composition of communities was significantly different between hotspots and coldspots and between sites, with a significant interaction between years and the nested factor site (Table 3). The two years 2008 and 2009 were not significantly different at any site (Table 4, Figure 4B). Dissimilarity within years was greater across coldspots than across hotspots and comparable in 2008 and 2009 (Table 5, Figure 4B).

### Influence of biotic and environmental factors on the variability in fluxes

**Figure 5:**
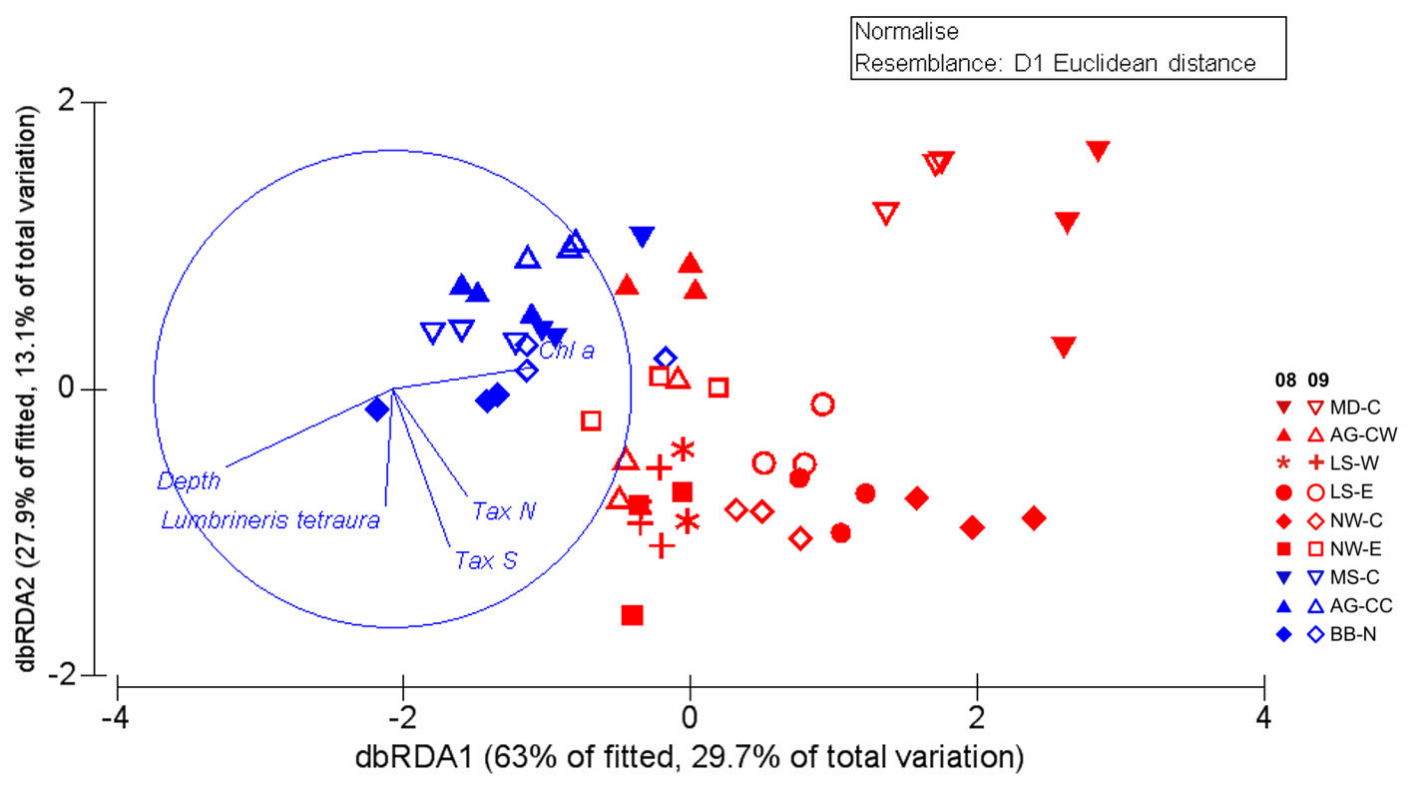
Distance-based Redundancy Analysis (dbRDA) plot of the distLM model based on the five parameters fitted to the variation in benthic boundary fluxes (Tables 6 & 7). Vectors indicate direction of the parameter effect in the ordination plot. Chl *a* = Ln of sediment Chl *a* concentration; N = abundance, Tax S = taxonomic richness. Red = hotspots, blue = coldspots; full symbols = 2008, open symbols = 2009; AG = Amundsen Gulf, MD = Mackenzie Delta, MS = Mackenzie Shelf/Slope; LS = Lancaster Sound; NW = North Water Polynya; = BB = Baffin Bay; C, E, N, W = central, east, north, west.

The best distance-based linear model (DistLM), explaining 42% of the overall variation in benthic boundary fluxes, was composed of five parameters (Figure 5, Table 6). Sediment surface Chl *a* concentration contributes most to the explained variation (23.5%), followed by S_Tax_ (9.5%), N (5.9%), water depth (4.3%) and abundance of *Lumbrineristetraura* (3.9%). Measures of functional diversity were not retained in the model. Variation of the first axis mainly separates coldspots from hotspots and pairs of the two years of each site (Figure 5). The most important parameters contributing to the first axis of the dbRDA plot, explaining 63% of fitted flux variation, are sediment surface Chl *a* concentration and water depth (Figure 5, Table 7). Benthic community parameters were most strongly correlated with the second dbRDA axis, explaining 27.9% of fitted flux variation (Table 7).

**Table 6:** Distance-based linear model (DistLM) of benthic boundary fluxes against environmental and diversity drivers determined in the Canadian Arctic in 2008 and 2009.

Sequential tests for stepwise model (Adj. R^2^ = 42%)							
Variable	AICc	SS(trace)	Pseudo-F	P	Prop.	Cum.	res. df
Chl *a*	75.693	62.191	15.946	0.0001	0.235	0.235	52
S_Tax_	70.782	25.168	7.226	0.0001	0.095	0.330	51
N	68.158	15.591	4.811	0.0021	0.059	0.388	50
Depth	66.673	11.344	3.688	0.0069	0.043	0.431	49
*L* *. tetraura*	65.34	10.423	3.566	0.0149	0.039	0.471	48

Proportion of variance in benthic boundary fluxes explained by environmental variables in stepwise sequential tests following AICc selection criterion. Chl a = sediment Chl a concentration, S_Tax_ = taxonomic richness, N = individual abundance, Depth = water depth, L. = Lumbrineris. 'Prop.' is the proportion of variance explained by each single variable, 'Cum.' is the cumulative proportion of variance explained by multiple variables.

**Table 7:** DistLM components of benthic boundary fluxes against environmental and diversity drivers determined in the Canadian Arctic in 2008 and 2009.

	Percentage of multivariate flux variation explained by individual axes				Relationships between dbRDA coordinate axes and orthonormal X variables (multiple partial correlations)				
	out of fitted model [%]		out of total variation [%]		Chl *a*	S_Tax_	N	Depth	*L* *. tetraura*
Axis	Ind.	Cum.	Ind.	Cum.					
1	63.01	63.01	29.65	29.65	0.591	0.239	0.314	-0.702	-0.031
2	27.87	90.88	13.12	42.77	0.092	-0.662	-0.452	-0.328	-0.492
3	5.92	96.8	2.79	45.56	0.201	0.636	-0.707	0.079	-0.221
4	3.19	100	1.5	47.06	0.359	0.025	0.392	0.516	-0.671

Proportion of variance in benthic boundary fluxes explained by the 4 axis retained in the DistLM (Table 6), and their relation to the explaining variables. Chl a = sediment Chl a concentration, S_Tax_ = taxonomic richness, N = individual abundance, Depth = water depth, L. = Lumbrineris. 'Ind.' is the percentage of explained variation by the individual axis, 'Cum.' is the cumulative percentage of variance explained by multiple axis.

## Discussion

In the Arctic, long-term prediction of the ecosystem function is often based on one-site one-year measures due to scarcity of data. Here, we focus on the different patterns of (non-directional) temporal and spatial variability in benthic remineralisation function, functional and taxonomic diversity and resource availability, which we consider important terms of error for long-term predictions. We discuss the role of variability in hotspot *vs* coldspot regimes and finally, we discuss the limitations of a statistical model integrating environmental and macrofaunal parameters to explain directional temporal and spatial variation in benthic remineralisation function in the Arctic.

Hypothesis 1 Benthic remineralisation function is significantly different between years at hotspots but not at coldspots

Simultaneous consideration of five benthic boundary fluxes confirmed that the magnitude of interannual variability in benthic remineralisation function differed between hotspots and coldspots. At hotspot sites the between-years differences were generally more pronounced than at coldspot sites, but the results are less consistent than we assumed. The composition of fluxes at different sites is heterogeneous due to complex interactions with environmental [22,72] and faunal [24,73] parameters. Change in benthic boundary fluxes from one year to another can be positive or negative in its direction, depending on the flux and on the site. This means that a given site x in 2008 can be different from the same site x in 2009, but similar to another site y in 2009 (e.g. LS-W and NW–C, Figures 2, 3A), both being hotspots. When remineralisation function as a whole is considered, it is therefore not surprising that the relative change from 2008 to 2009 across all hotspots or all coldspots is not significant, although differences between years are found for four of six hotspots. In fact, the interannual differences at the remaining two hotspots might not be detectable due to the strong within-site variation, but they show the tendency of a shift. The coldspot site BB–N was not only different in 2008 than in 2009, but also differed from other coldspot fluxes. High nitrite uptake of about 30 µmol m^-2^ d^-1^ may be the underlying mechanism. In this study, such high nitrite uptake has only been found at hotspots with other high fluxes (NW-E and LS-E) and typically indicates bacteria-mediated anaerobic degradation of nitrogen derivates such as ammonium oxidation (anammox). Anammox is considered a common process in deep Arctic cold-water environments, and the nitrite uptake rates measured in 2008 might be a lag response to the degradation of intensive organic matter pulses fuelling abundance and degradation by anammox bacteria [74]. Site-dependent changes in nutrient fluxes were also found in a shallow-water environment by Thouzeau et al. [75], who interpreted interannual differences of biogeochemical fluxes as being mostly affected by differences in environmental parameters and organic matter deposition, including indirect effects of macrofauna and macroalgae, depending on the sites. Similar factors could have affected the changes at our hotspot sites.

One of the rare time-series studies including the measurement of total sediment oxygen fluxes in deeper water was conducted in the abyssal north Pacific for eight years [76]. Although the export of organic matter varied between years, oxygen consumption remained fairly stable. Smith et al. [76] interpreted this discrepancy in the rather oligotrophic environment they investigated as a capacity of the benthic fauna to endure food deficiency over a limited time period – until a new food pulse arrives. Such an explanation would also fit to oxygen flux patterns of coldspots in our study. In a less oligotrophic environment, Lepore et al. [77] compared sediment oxygen fluxes (expressed as benthic carbon respiration) between years in the Chukchi Sea in 2002 and 2004. Summed over several sites, they found that benthic carbon respiration did not change much, neither on the shelf nor on the slope over the years, despite great changes in vertical carbon export. One possible explanation for this finding is a time lag in the response of the sediment communities. However, across-site patchiness in benthic carbon respiration was high and may have masked temporal variations at particular sites – as it was the case across hotspots and coldspots in our study. This stresses the importance to separate spatial from temporal variability if we want to understand dynamics of ecosystem functions. A few sites in our study have been sampled prior to 2008. In 2004, Renaud et al. [78] reported less than half the oxygen demand (5.65 mmol m^-2^ d^-1^) of our values at hotspot site MD-C but similar values at hotspot site AG-CW (2.12 mmol m^-2^ d^-1^). At hotspot site NW–C, the oxygen fluxes we measured in 2008 were twice as high as those measured in 1998 (4.3 mmol m^-2^ d^-1^ [53]), but they were largely the same in 2009. Longer time-series measurements are necessary to draw a firm conclusion, but these results may indicate that a progressive change is already happening in the Mackenzie Delta region, whereas there is stochastic variability in the NOW region.

We found that differences between sites can be in the same order of magnitude as variations across years (Figure 3A). However, the significant difference in remineralisation function between hotspots and coldspots statistically confirms our *a priori* categorisation of sites. Although we cannot separate a location from dispersion effect [70] in the data, our results clearly showed that benthic remineralisation function is more variable between years at hotspot sites than at coldspot sites. This means that quantifying progressive directional changes in ecosystem functions at hotspot sites requires a long-term data series [34], whereas coldspot sites might require less regular sampling to detect changes.

Hypothesis 2 Food availability is significantly different between years at hotspots but not at coldspots

Only one of six hotspot sites but two of three coldspot sites changed significantly over the years, but our results showed that the magnitude of interannual variations in food supply to the benthos, as approximated by sediment pigment concentrations, differed between hotspots and coldspots and depends on the considered site. Also, hotspots were different from coldspots. How can we explain the unexpectedly rare interannual changes at hotspots and more common changes at coldspots? Here, we analysed concentration of rather labile Chl *a* and stable, more degraded phaeopigments in the sediments simultaneously. Phaeopigments can accumulate with degradation of matter and are therefore not necessarily an indicator of recent organic matter input, which we want to detect when looking at annual variability. But since Chl *a* is often rapidly degraded to phaeopigments [79], the latter allow the detection of food input even if it had been of lower quality or earlier in the year. Another indicator of food at the seafloor is the ratio of Chl *a* over phaeopigment concentration (e.g. [80]). But as a quality ratio, this measure does not allow the comparison of the quantity of pigments at different sites. Since the quality of food is often related to the activity of benthic communities [29,79], we could consider only using sediment Chl *a* concentration, which should be representative of fresh matter input having occurred in the same season. However, statistical analyses using only sediment Chl *a* did not yield very different results, and only hotspot MS-C differed significantly between years (data not shown). Furthermore, one important issue that could make changes statistically less detectable is the small-scale spatial (within-site) variation in our sediment pigment data [70]. Furthermore, dispersion was greater across hotspot sites than across coldspot sites, impeding the detection of changes in the plot. In fact, the results from the SIMPER analysis clearly showed that hotspots were more different from one year to another than coldspots, but also that the difference between years for coldspots was large compared to the variability between coldspots of the same year.

Interannual changes of sediment pigments were found at several depths in the Fram Strait at the HAUSGARTEN site in Norway [35], which could be related to a decrease in the vertical flux of phytodetrital matter. In our study area, interannual variability of vertical flux patterns is only known for part of the study period and study area. In the southeastern Beaufort Sea, Sallon et al. [81] found two-day vertical fluxes at hotspots MS-C and AG-CW in 2008 that were similar to those reported in 2004 [82]. Both were higher than those determined in the 1980s [42]. Recent results from 2009 showed, however, that vertical fluxes at theses sites were lower than in 2008 [22,83]. Due to the generally tight pelagic-benthic coupling in the Arctic, such interannual variability in vertical fluxes in the southeastern Beaufort Sea (see also 84 for a more regional approach) could lead to interannual sediment pigment changes, even at coldspots. In the NOW, at the hotspots NW–C and NW-E, Hargrave et al. [52] showed an about two-fold increase of vertical flux from 1998 to 1999. In the LS-W region, strong interannual variability of processes determining pelagic-benthic coupling, including the release of ice algae, have been observed between 1984 and 1992 [44]. However, in that period the change seemed stochastic, and contrarily we may have sampled in two years of similar vertical export in 2008-2009. Although the different studies on all the subregions show interannual variability, we do not know how or whether vertical fluxes changed between 2008 and 2009, and it is therefore difficult to explain the lack of signal for sediment pigments using vertical flux patterns. In the area of LS-E or BB–N, no data on vertical fluxes has been published to our knowledge, thus that we can only infer variability from primary production and phytoplankton Chl *a* biomass patterns. Significant interannual differences between 2005 and 2007 (confounded with season late summer, early fall and fall) in primary production have not been found for the Beaufort Sea, Archipelago (including Lancaster Sound) or NOW [37]. However, phytoplankton Chl *a* biomass at individually resampled sites AG-CW (408), AG-CC (405), NW–C (108) and NW-E (115) seemed different in 2005 than in 2006 and 2007. But at water depths of more than 200 m, several processes influence the actual export of primary production to the seafloor. Temporal and spatial variability in these pelagic processes may nullify or even invert an increase in primary production to a decrease in vertical export, as has been reported at some sites in the southeastern Beaufort Sea in 2008 [28,81]. Such discrepancy is particularly evident for site AG-CC, which Ardyna et al. [37] identified as a hotspot of primary production, but which is consistently a coldspot in benthic parameters. Lateral advection has been proposed as mechanism underlying discrepancies between surface primary production and benthic activity [77]. However, currents in the central Amundsen Gulf region are generally weak, thus that pelagic degradation is probably more important [28,84]. Extrapolating temporal and spatial changes in benthic food supply from primary production patterns should be treated with extreme caution.

Benthic degradation of organic matter could theoretically also be the reason why coldspots with consistently lower sediment pigment concentration demonstrate more changes than hotspots: Assuming that hotspots generally host higher abundance of organisms due to generally higher food supply (e.g. [85]), such higher density could consume a peak of arriving food more rapidly than a site with lower abundance. In our study, surface deposit feeders were more abundant at hotspot sites (average of 120 per site) than at coldspot sites or sites with significant differences in food supply between years (average of 10 or 12 per site, respectively) (data not shown). Rapid consumption of organic material has been demonstrated for seafloor communities of different depths [86] and Arctic locations [29,79]. If sediment food supply is measured months after the input peak, the measured sediment pigments could represent the ‘leftovers of the feast’, which should vary less when abundance is high. In this case, food supply may work better as proxy for benthic functions, where rapid deposit feeding groups are absent. It is also interesting to note, that average similarity of functional community composition between years is lower at sites with significant differences in food supply between 2008 and 2009 (Table 4). Overall, the results indicate that functional community composition may bias food supply proxies. Hence, when explaining changes in benthic function, the interaction of community composition on measured food supply (i.e. the remaining stock) could influence the results.

Hypothesis 3 Taxonomic community composition is not significantly different between years

Macrobenthic communities at hotspots or coldspots in the Canadian Arctic did not change significantly in taxonomic composition from 2008 to 2009. Instead, the community composition is generally different at hotspots than that at coldspots, and sites are distinct from each other. The total number of 331 macrobenthic taxa found in our samples may seem low compared to the overall number of 992 taxa reported from the entire Canadian Arctic [87]. Piepenburg et al. [13] performed a rarefaction analysis based on molluscs, arthropods, echinoderms and annelids for different regions in the Arctic. They showed that 19 sampling events would yield an average of 274 observed taxa in the Amundsen Gulf region and 205 on the Beaufort Shelf. Moreover, based on fewer stations, totals of 86 and 204 taxa have been reported for Lancaster Sound and northern Baffin Bay, respectively [13]. Considering species overlap between the different regions and a total of 18 sampling events, we can assume to have taken and analysed a typical number of species for the sampling size. The number of taxa we found at sites in the NOW region was also lower than reported by Lalande [54]. But due to the different sampling approaches applied (here: 3 incubation cores of 80 cm^2^ each *vs* large corers covering areas of usually 1250 cm^2^), our results are not directly comparable, and the numbers most likely only represent differences in total sampled surface. Two data distribution patterns in the MDS plot (Figure 4A) are interesting to note: First, with the exception of site AG-CW 2008, hotspot sites are well separated from coldspot sites. This observation supports the notion that species composition is largely dependent on regimes of food supply [88], since primary production regimes were part of our hotspot definition. Second, sites are clustered according to their depth within the hotspot or coldspot groups (which is much less the case for benthic fluxes) (Figure 4A). This indicates depth zonation of benthic infauna communities on a shelf gradient, which has previously been reported [43,89], but could not be identified by Cusson et al. [12] in other regions of the Canadian Arctic. These results of within-regime gradients stress the importance that environmental factors other than depth create communities of different diversity and composition.

In contrast to spatio-temporal patterns in benthic remineralisation function and food supply, the significant interaction between the factors year and site in our data results only from statistically different pairs of different sites across years. In support of this, similar variability of communities across sites in 2008 and 2009 is confirmed through the SIMPER analysis. Polar environments host species with longer life spans, and community turnover rates should therefore be slower. In an Arctic Fjord, Kedra et al. [63] reported a decadal shift in communities in two of the fjord regions influenced by an intrusion of warm water, but no changes in a third region with more stable hydrographic conditions. This supports the notion that community changes are rather long-term reactions to environmental forcing. Similarly, long-term time series of macrobenthic infauna in the northern Bering Sea show progressive change in community composition since 2000 on a decadal scale, although the change is not yet statistically detectable [32]. At sites in Frobisher Bay (Canadian Arctic), however, Cusson et al. [12] detected monthly variability in community composition. Considering that those communities became similar over time again, the effect of lacking replicate data information could have reduced both within-month and within-site variability and thus produced an artificial signal. The overall low within-site variability of Frobisher Bay sites compared to other sites in the Canadian Arctic supports this explanation. Interannual variations in meiofaunal abundance were found during time-series measurements at the HAUSGARTEN from 2000–2004 [89]. However, these changes were less clear when community composition was considered. Overall, the results support our hypothesis and spatial variability in community composition seems more pronounced than interannual variations. However, without separating the temporal from the spatial component of variability, we will miss detecting long-term shifts in community composition, such as the shift reported by Grebmeier [32] in the northern Bering Sea.

Hypothesis 4 Functional community composition is not significantly different between years

Our results support the hypothesis that macrobenthic communities at hotspots or coldspots in the Canadian Arctic did not change significantly in functional composition from 2008 to 2009. Instead, the functional community composition is generally different at hotspots than that at coldspots, and sites are distinct from each other. The number of 72 functional groups reported in our study may seem very high, but if functional groups are to be related to ecosystem functions, it is crucial to include as many different trait categories as known to influence those functions [60]. We therefore think that such fine-scale categorisation is adequate. Functional richness (number of functional groups) was largely similar to the number of taxa at sites with only few taxa, but was clearly lower at sites with many taxa (Table S2). This is a typical phenomenon, since in small-scale studies, the probability to find species redundant in their functions increases with the total number of species encountered [90]. Functional diversity can follow changes in taxonomic diversity – or not [91,92]. If functional redundancy is present (as for samples where taxonomic diversity is higher than functional diversity), a reduction in the number of species will not necessarily decrease the number of functional groups. This implies less variability, and the absence of changes in functional composition over the years matches well with temporal patterns in taxonomic composition.

When comparing results of variation in functional composition and taxonomic composition, we again find a well-defined separation of hotspot and coldspot sites. However, sites are generally more similar to each other when functional diversity is analysed. Using functional diversity in comparative studies has received increasing attention over the last decades [2]. An important advantage of this approach is its lower susceptibility to misclassifications, and Cochrane et al. [93] have recently demonstrated its utility to describe ecologically distinct regions in the Barents Sea. Although the general power to identify spatial and temporal differences in community composition is reduced when the functional (opposed to taxonomic) approach is used, functional diversity is easier to use and sufficient to describe the ecological role of different communities.

Hypothesis 5 Food supply explains temporal variation and macrofaunal community parameters explain spatial variation in benthic remineralisation function

The results of this study confirm our hypothesis that the temporal variability of benthic remineralisation function is most affected by sediment pigments while its spatial variation is largely determined by diversity patterns. However, water depth was an additional important factor explaining spatial patterns, both across sites and between hotspots and coldspots. We demonstrated how depth played a role in explaining the resemblance patterns in taxonomic community composition (see section Hypothesis 3). Moreover, water depth has been used as a rough estimate for food supply to the benthos [94], as also indicated by its similar relationship to the variation axes as that of sediment Chl *a* concentrations in our data. The importance of an easy-to-determine and steady-state variable for ecosystem functions can have stabilizing effects in predicting models, but also calls for caution considering its low explicative power and when ecosystems of different depths and regimes are compared [34]. In our data, sediment Chl *a* concentrations are strongly related to the variation axis across which hot- and coldspots are separated, and along which temporal variability of sites is spread, despite the lack of temporal variability in the variable itself. The retention of Chl *a* but not phaeopigments in our model underpins the notion that fresh food supply rather than general food supply is most important in determining benthic remineralisation function [22,29,79].

About 20% of the variation in benthic function is explained by different measures of diversity (richness of taxa, individual abundance, and the abundance of *L* *. tetraura* ). These three variables mostly explain differences between hotspots and coldspots and the eastern and western Canadian Arctic, but also temporal variation at hotspots AG-CW and NW-E. The better explicative power of taxonomic *vs* functional group richness may be due to several mechanisms. When relating functional diversity to ecosystem functions, species traits relevant for the considered ecosystem functions need to be included [60]. Bioirrigation has been shown to influence biogeochemical fluxes across the sediment-water interface [73,95,96]. The lack of information for species in our study did not allow including this trait as a category, but including traits more directly linked to functions, in our case e.g. including bioirrigation may improve the explanatory power of functional type models for ecosystem functions. Further, resource availability, measured as Chl *a* in our study, may affect the diversity-ecosystem function relationship. At large spatial scales across the marine Arctic, the number of benthic species generally increases with primary production, if the effect of salinity is removed from the model [97]. If functional group richness is more related to sediment Chl *a* concentration, then taxonomic richness is chosen as a more complementary variable to the already used Chl *a* in the model. Moreover, interactions between functional and species richness in their effect on functions have been found, making species richness more important, if functional richness is low [10]. Another possible cause for taxonomic richness being more important is the use of multiple processes defining our function: Using multiple processes decreases the chance that several species are redundant in effecting the functions [98].

Our statistical approach of a predictive linear model does not allow testing the combined effect of food supply and whole community composition on multiple benthic fluxes. But total abundance and the abundance of a gallery-burrowing polychaete species as significant predictors for multiple benthic functions stress the importance of community composition additionally to mere species numbers. In fact, the density of fauna [99], identity or functional traits of species [24,25] and the number of burrows at the seafloor [73] have been related to benthic boundary fluxes before.

More than half of the variation in benthic boundary fluxes in our data could not be explained by our best model. As already mentioned above, more exact measures of community composition or trait composition could explain more variation. Two other important benthic community components are also lacking in our study: meiobenthic and bacterial abundance. The role of meiobenthos for biogeochemical cycles is less studied, but there is increasing evidence that its density is related to benthic remineralisation function [25,100,101]. The role of bacteria is known to be relevant for organic matter degradation processes [102]. Different types of bacteria can influence the particular process of degradation (e.g. anammox), can react rapidly to matter input and interact with macrofaunal matter degradation processes [74,103,104]. Although evidence is increasing that macrofauna drives the variability in bacteria-mediated degradation [104,105], future studies should integrate meiofaunal and microbenthic organisms to gain a better understanding of mechanisms regulating temporal and spatial variability of benthic remineralisation function.

Benthic ecosystem function in the Canadian Arctic increases mostly with fresh food supply, species-rich and functional diverse communities. With climate change, the quantity and - even more importantly - the quality of food supply to polar benthos are changing [14,28,30]. While we currently still know only little about how benthic communities will react to these changes [32], we could show here that diversity changes will also have an impact on the quality of benthic ecosystem functions. As recently indicated in a metaanalysis on studies from the aquatic environment [8], our study confirms the comparable importance of abiotic factors and diversity for ecosystem functions in a natural marine environment.

## Conclusion

Great efforts are underway to estimate the impact of climate change on polar ecosystems. Due to the very limited number of real benthic time-series measurements in the Arctic, ecosystem models often rely on data obtained from different sites at different times of the year. One important question under these circumstances is: Are known benthic diversity hotspots in the Canadian Arctic also hotspots in ecosystem function? We conclude from our findings: Yes, with regard to a general spatial comparison, but No, with regard to long-term predictions, which may increase variability of resource availability due to climate-related ecological change. In our study, we have demonstrated that the influence of diversity on multiple benthic ecosystem functions is complementary or dependent on the availability of resources. The mechanisms controlling temporal variability of factors explaining benthic ecosystem function vary even on a within-region spatial scale. We have also shown that the similarity in taxonomic and functional diversity patterns indicate little insurance against climate induced species loss in the Canadian Arctic. Even models that include several variables (steady-state ones but also those varying on short and long time scales) explained only half of the variability in multiple benthic ecosystem function in the Canadian Arctic. Defining the role of the functional identity of particular organisms in benthic biogeochemical cycles should help to better predict benthic remineralisation function. Our findings also strongly suggest that for reliable predictions of how ecosystem functioning in Arctic shelf habitats will change in the future and how close we are to tipping points, it is necessary to establish time-series sites at hotspots and coldspots where multiple function measures are monitored, in order to distinguish natural oscillations from directional change.

## Supporting Information

Table S1 **Taxa list.** The table presents all taxa identified during this study and the accorded functional traits.
(PDF)

Table S2 **Sediment pigment concentrations (Chl *a* and phaeopigments ‘Phaeo’), community descriptors (taxonomic richness S_Tax_, total abundance N, functional group richness S_Func_ and Shannon-Wiener Index H'_Func_) and abiotic variables used in the study.**
(PDF)

## References

[1] Naeem S, Bunker DE, Hector A, Loreau M, Perrings C (2009) Introduction: The ecological and social implications of changing biodiversity. An overview of a decade of biodiversity and ecosystem functioning research. In: Naeem S Bunker DE Hector A Loreau M Perrings C Biodiversity, Ecosystem Functioning, and Human Wellbeing: An Ecological and Economic Perspective. Oxford, UK: Oxford University Press pp. 3-13.

[2] Naeem S, Duffy JE, Zavaleta E (2012) The Functions of Biological Diversity in an Age of Extinction. Science 336: 1401-1406. doi:10.1126/science.1215855. PubMed: 22700920. 22700920 10.1126/science.1215855

[3] Schmid B, Balvanera P, Cardinale B, Godbold J, Pfisterer AB et al. (2009) Consequences of species loss for the ecosystem functioning: meta-analyses of data from biodiversity experiments. In: Naeem S Bunker DE Hector A Loreau M Perrings C Biodiversity, Ecosystem Functioning, and Human Wellbeing: An Ecological and Economic Perspective. Oxford, UK: Oxford University Press pp. 14-29.

[4] Cardinale BJ, Duffy JE, Gonzalez A, Hooper DU, Perrings C et al. (2012) Biodiversity loss and its impact on humanity. Nature 486: 59-67. doi:10.1038/nature11148. PubMed: 22678280. 22678280 10.1038/nature11148

[5] Hooper DU, Adair EC, Cardinale BJ, Byrnes JEK, Hungate BA et al. (2012) A global synthesis reveals biodiversity loss as a major driver of ecosystem change. Nature 486: 105-U129. PubMed: 22678289. 22678289 10.1038/nature11118

[6] Myers N, Mittermeier RA, Mittermeier CG, da Fonseca GAB, Kent J (2000) Biodiversity hotspots for conservation priorities. Nature 403: 853-858. doi:10.1038/35002501. PubMed: 10706275. 10706275 10.1038/35002501

[7] Fridley JD (2002) Resource availability dominates and alters the relationship between species diversity and ecosystem productivity in experimental plant communities. Oecologia 132: 271-277. doi:10.1007/s00442-002-0965-x. 28547362 10.1007/s00442-002-0965-x

[8] Godbold JA (2012) Effects of biodiversity-environment conditions on the interpretation of biodiversity-function relations. In: Solan M Aspden RJ Paterson DM Marine Biodiversity and Ecosystem Functioning : Frameworks, Methodologies, and Integration. Oxford, GBR. Oxford University Press pp. 101-114.

[9] Godbold JA, Solan M (2009) Relative importance of biodiversity and the abiotic environment in mediating an ecosystem process. Mar Ecol Prog S 396: 273-282. doi:10.3354/meps08401.

[10] Wahl M, Link H, Alexandridis N, Thomason JC, Cifuentes M et al. (2011) Re-Structuring of Marine Communities Exposed to Environmental Change: A Global Study on the Interactive Effects of Species and Functional Richness. PLOS ONE 6: e19514. doi:10.1371/journal.pone.0019514. PubMed: 21611170. 21611170 10.1371/journal.pone.0019514 PMC3097188

[11] Doney SC, Ruckelshaus M, Emmett Duffy J, Barry JP, Chan F et al. (2012) Climate Change Impacts on Marine Ecosystems. Annu Rev Mar Science 4: 11-37. doi:10.1146/annurev-marine-041911-111611. PubMed: 22457967. 10.1146/annurev-marine-041911-111611 22457967

[12] Cusson M, Archambault P, Aitken A (2007) Biodiversity of benthic assemblages on the Arctic continental shelf: historical data from Canada. Mar Ecol Prog S 331: 291-304. doi:10.3354/meps331291.

[13] Piepenburg D, Archambault P, Ambrose JW, Blanchard A, Bluhm B et al. (2011) Towards a pan-Arctic inventory of the species diversity of the macro- and megabenthic fauna of the Arctic shelf seas. Mar Biodivers 41: 51-70. doi:10.1007/s12526-010-0059-7.

[14] Wassmann P, Duarte CM, Agusti S, Sejr MK (2011) Footprints of climate change in the Arctic marine ecosystem. Glob Change Biol 17: 1235-1249. doi:10.1111/j.1365-2486.2010.02311.x.

[15] Emmerson MC, Solan M, Emes C, Paterson DM, Raffaelli D (2001) Consistent patterns and the idiosyncratic effects of biodiversity in marine ecosystems. Nature 411: 73-77. doi:10.1038/35075055. PubMed: 11333979. 11333979 10.1038/35075055

[16] Danovaro R (2012) Extending he approaches of biodiversity and ecosystem functioning to the deep ocean. In: Solan M Aspden RJ Paterson DM Marine Biodiversity and Ecosystem Functioning: Frameworks, Methodologies, and Integration. Oxford, GBR. Oxford University Press pp. 115-126.

[17] Clough LM, Renaud PE, Ambrose WG (2005) Impacts of water depth, sediment pigment concentration, and benthic macrofaunal biomass on sediment oxygen demand in the western Arctic Ocean. Can J Fish Aquat Sci 62: 1756-1765. doi:10.1139/f05-102.

[18] Grebmeier JM, Cooper LW, Feder HM, Sirenko BI (2006) Ecosystem dynamics of the Pacific-influenced Northern Bering and Chukchi Seas in the Amerasian Arctic. Prog Oceanogr 71: 331-361. doi:10.1016/j.pocean.2006.10.001.

[19] Renaud PE, Riedel A, Michel C, Morata N, Gosselin M et al. (2007) Seasonal variation in benthic community oxygen demand: A response to an ice algal bloom in the Beaufort Sea, Canadian Arctic? J Mar Syst 67: 1-12. doi:10.1016/j.jmarsys.2006.07.006.

[20] Darnis G, Robert D, Pomerleau C, Link H, Archambault P et al. (2012) Current state and trends in Canadian Arctic marine ecosystems: II. Heterotrophic food web, pelagic-benthic coupling, and biodiversity. Clim Change 115: 179-205. doi:10.1007/s10584-012-0483-8.

[21] Robert P, McKindsey CW, Chaillou G, Archambault P (2013) Dose-dependent response of a benthic system to biodeposition from suspended blue mussel (Mytilus edulis) culture. Mar Pollut Bull 66: 92-104. doi:10.1016/j.marpolbul.2012.11.003. PubMed: 23219398. 23219398 10.1016/j.marpolbul.2012.11.003

[22] Link H, Chaillou G, Forest A, Piepenburg D, Archambault P (2012) Multivariate benthic ecosystem functioning in the Arctic – benthic fluxes explained by environmental parameters in the southeastern Beaufort Sea. Biogeosciences Discuss 9: 16933-16976. doi:10.5194/bgd-9-16933-2012.

[23] Ieno EN, Solan M, Batty P, Pierce GJ (2006) How biodiversity affects ecosystem functioning: roles of infaunal species richness, identity and density in the marine benthos. Mar Ecol Prog S 311: 263-271. doi:10.3354/meps311263.

[24] Michaud E, Desrosiers G, Mermillod-Blondin F, Sundby B, Stora G (2006) The functional group approach to bioturbation: II. The effects of the Macoma balthica community on fluxes of nutrients and dissolved organic carbon across the sediment-water interface. J Exp Mar Biol Ecol 337: 178-189. doi:10.1016/j.jembe.2006.06.025.

[25] Piot A (2012) Impacts des changements de biodiversité sur le fonctionnement de l’écosystème benthique en zone intertidale. Rimouski: Université du Québec à Rimouski.

[26] Harvey E, Séguin A, Nozais C, Archambault P, Gravel D (2012) Identity effects dominate the impacts of multiple species extinctions on the functioning of complex food webs. Ecology 94: 169-179. PubMed: 23600251. 10.1890/12-0414.1 23600251

[27] Kenchington E, Link H, Roy V, Archambault P, Siferd T et al. (2011) Identification of Mega- and Macrobenthic Ecologically and Biologically Significant Areas (EBSAs) in the Western, Central and Eastern Canadian Arctic. Department of Fisheries and Oceans Canada. Res. Doc. 2011/071. iv, 52 p

[28] Forest A, Tremblay J-É, Gratton Y, Martin J, Gagnon J et al. (2011) Biogenic carbon flows through the planktonic food web of the Amundsen Gulf (Arctic Ocean): A synthesis of field measurements and inverse modeling analyses. Prog Oceanogr 91: 410-436. doi:10.1016/j.pocean.2011.05.002.

[29] Link H, Archambault P, Tamelander T, Renaud PE, Piepenburg D (2011) Spring-to-summer changes and regional variability of benthic processes in the western Canadian Arctic. Polar Biol 34: 2025-2038. doi:10.1007/s00300-011-1046-6.

[30] Tremblay J-É, Bélanger S, Barber D, Asplin M, Martin J et al. (2011) Climate forcing multiplies biological productivity in the coastal Arctic Ocean. Geophys Res Lett 38: L18604.

[31] Baldwin RJ, Glatts RC, Smith KL Jr (1998) Particulate matter fluxes into the benthic boundary layer at a long time-series station in the abyssal NE Pacific: composition and fluxes. Deep Sea Res II Topical Stud Oceanogr 45: 643-665. doi:10.1016/S0967-0645(97)00097-0.

[32] Grebmeier JM (2012) Shifting Patterns of Life in the Pacific Arctic and Sub-Arctic Seas. Annu Rev Mar Science 4: 63-78. doi:10.1146/annurev-marine-120710-100926. PubMed: 22457969. 10.1146/annurev-marine-120710-100926 22457969

[33] Glud RN, Risgaard-Petersen N, Thamdrup B, Fossing H, Rysgaard S (2000) Benthic carbon mineralization in a high-Arctic sound (Young Sound, NE Greenland). Mar Ecol Prog S 206: 59-71. doi:10.3354/meps206059.

[34] Glover AG, Gooday AJ, Bailey DM, Billett DSM, Chevaldonné P et al. (2010) One - Temporal Change in Deep-Sea Benthic Ecosystems: A Review of the Evidence From Recent Time-Series Studies. In: Michael L Advances in Marine Biology. Academic Press pp. 1-95. 10.1016/B978-0-12-381015-1.00001-0 20959156

[35] Soltwedel T, Bauerfeind E, Bergmann M, Budaeva N, Hoste E et al. (2005) HAUSGARTEN: multidisciplinary investigations at a deep-sea, long-term observatory in the Arctic Ocea. Oceanography 18: 46-61. doi:10.5670/oceanog.2005.24.

[36] Sakshaug E (2004) Primary and Secondary Production in the Arctic Seas. In: Stein R Macdonald RW The Organic Carbon Cycle in the Arctic Ocean. Berlin Heidelberg, Germany: Springer pp. 57-82.

[37] Ardyna M, Gosselin M, Michel C, Poulin M, Tremblay JE (2011) Environmental forcing of phytoplankton community structure and function in the Canadian High Arctic: contrasting oligotrophic and eutrophic regions. Mar Ecol Prog S 442: 37-57. doi:10.3354/meps09378.

[38] Arrigo KR, van Dijken GL (2004) Annual cycles of sea ice and phytoplankton in Cape Bathurst polynya, southeastern Beaufort Sea, Canadian Arctic. Geophys Res Lett 31: L08304. doi:10.1029/2003GL018978.

[39] Mundy CJ, Gosselin M, Ehn J, Gratton Y, Rossnagel A et al. (2009) Contribution of under-ice primary production to an ice-edge upwelling phytoplankton bloom in the Canadian Beaufort Sea. Geophys Res Lett 36: L17601. doi:10.1029/2009GL038837.

[40] Forest A, Sampei M, Hattori H, Makabe R, Sasaki H et al. (2007) Particulate organic carbon fluxes on the slope of the Mackenzie Shelf (Beaufort Sea): Physical and biological forcing of shelf-basin exchanges. J Mar Syst 68: 39-54. doi:10.1016/j.jmarsys.2006.10.008.

[41] Lalande C, Forest A, Barber DG, Gratton Y, Fortier L (2009) Variability in the annual cycle of vertical particulate organic carbon export on Arctic shelves: Contrasting the Laptev Sea, Northern Baffin Bay and the Beaufort Sea. Contin Shelf Res 29: 2157-2165. doi:10.1016/j.csr.2009.08.009.

[42] O’Brien MC, Macdonald RW, Melling H, Iseki K (2006) Particle fluxes and geochemistry on the Canadian Beaufort Shelf: Implications for sediment transport and deposition. Contin Shelf Res 26: 41-81. doi:10.1016/j.csr.2005.09.007.

[43] Conlan K, Aitken A, Hendrycks E, McClelland C, Melling H (2008) Distribution patterns of Canadian Beaufort Shelf macrobenthos. J Mar Syst 74: 864-886. doi:10.1016/j.jmarsys.2007.10.002.

[44] Michel C, Ingram RG, Harris LR (2006) Variability in oceanographic and ecological processes in the Canadian Arctic Archipelago. Prog Oceanogr 71: 379-401. doi:10.1016/j.pocean.2006.09.006.

[45] Welch HE, Siferd TD, Bruecker P (1997) Marine zooplanktonic and benthic community respiration rates at Resolute, Canadian high Arctic. Can J Fish Aquat Sci 54: 999-1005.

[46] Fortier M, Fortier L, Michel C, Legendre L (2002) Climatic and biological forcing of the vertical flux of biogenic particles under seasonal Arctic sea ice. Mar Ecol Prog S 225: 1-16. doi:10.3354/meps225001.

[47] Thomson D (1982) Marine Benthos in the Eastern Canadian High Arctic: Multivariate Analyses of Standing Crop and Community Structure. Arctic 35: 61-74.

[48] McLaughlin FA, Carmack EC, Ingram RG, Williams W, Michel C (2006) Oceanography of the North West Passage. In: Robinson AR Brink KH The Global Coastal Ocean - Interdisciplinary Regional Studies and Synthesis. Cambridge, USA: Harvard University Press pp. 163-192.

[49] Dumont D, Gratton Y, Arbetter TE (2009) Modeling the Dynamics of the North Water Polynya Ice Bridge. J Phys Oceanogr 39: 1448-1461. doi:10.1175/2008JPO3965.1.

[50] Ingram RG, Bacle J, Barber DG, Gratton Y, Melling H (2002) An overview of physical processes in the North Water. Deep Sea Res II Topical Stud Oceanogr 49: 4893-4906. doi:10.1016/S0967-0645(02)00169-8.

[51] Klein B, LeBlanc B, Mei Z-P, Beret R, Michaud J et al. (2002) Phytoplankton biomass, production and potential export in the North Water. Deep Sea Res II Topical Stud Oceanogr 49: 4983-5002. doi:10.1016/S0967-0645(02)00174-1.

[52] Hargrave BT, Walsh ID, Murray DW (2002) Seasonal and spatial patterns in mass and organic matter sedimentation in the North Water. Deep Sea Res II Topical Stud Oceanogr 49: 5227-5244. doi:10.1016/S0967-0645(02)00187-X.

[53] Grant J, Hargrave B, MacPherson P (2002) Sediment properties and benthic-pelagic coupling in the North Water. Deep Sea Res II Topical Stud Oceanogr 49: 5259-5275. doi:10.1016/S0967-0645(02)00189-3.

[54] Lalande C (2003) Composition et Structure de la Communauté Benthique et Quantification de la Bioturbation dans la Polynie des Eaux du Nord. Rimouski: Université du Québec à Rimouski.: p. 59.

[55] Deming J, Fortier L (2011) Introduction to the special issue on the biology of the circumpolar flaw lead (CFL) in the Amundsen Gulf of the Beaufort Sea (Arctic Ocean). Polar Biol 34: 1797-1801. doi:10.1007/s00300-011-1125-8.

[56] Snelgrove PVR, Archambault P, Juniper K, Lawton P, Metaxas A et al. (2012) Canadian Healthy Oceans Network (CHONe): An Academic–Government Partnership to Develop Scientific Guidelines for Conservation and Sustainable Usage of Marine Biodiversity. Fisheries 37: 296-304. doi:10.1080/03632415.2012.696002.

[57] Riaux-Gobin C, Klein B (1993) Microphytobenthic Biomass Measurement Using HPLC and Conventional Pigment Analysis. In: Kemp P Sherr B Sherr E Cole J Handbook of methods in aquatic microbial ecology. Boca Raton: Lewis Publishers pp. 369-376.

[58] Bremner J, Rogers SI, Frid CLJ (2003) Assessing functional diversity in marine benthic ecosystems: a comparison of approaches. Mar Ecol Prog S 254: 11-25. doi:10.3354/meps254011.

[59] Pearson TH (2001) Functional group ecology in soft-sediment marine benthos: The role of bioturbation. Oceanography Mar Biol, Vol 39: 233-267.

[60] Petchey OL, Gaston KJ (2006) Functional diversity: back to basics and looking forward. Ecol Lett 9: 741-758. doi:10.1111/j.1461-0248.2006.00924.x. PubMed: 16706917. 16706917 10.1111/j.1461-0248.2006.00924.x

[61] Appeltans W, Bouchet P, Boxshall GA, De Broyer C, de Voogd NJ et al. (eds) (2012) World Register of Marine Species. Available: http://www.marinespecies.org. Accessed 2012 July 28.

[62] Fauchald K, Jumars PA (1979) The diet of worms: A study of polychaete feeding guilds. Oceanogr Mar Biol Annu Rev 17: 193-284.

[63] Kedra M, Wlodarska-Kowalczuk M, Weslawski JM (2010) Decadal change in macrobenthic soft-bottom community structure in a high Arctic fjord (Kongsfjorden, Svalbard). Polar Biol 33: 1-11. doi:10.1007/s00300-009-0679-1.

[64] MacDonald IR, Bluhm BA, Iken K, Gagaev S, Strong S (2010) Benthic macrofauna and megafauna assemblages in the Arctic deep-sea Canada Basin. Deep Sea Res II Topical Stud Oceanogr 57: 136-152. doi:10.1016/j.dsr2.2009.08.012.

[65] MarLIN (2006) BIOTIC - Biological Traits Information Catalogue. In: Network MLI, editor; Plymouth: Marine Biological Association of the United Kingdom. Available: http://www.marlin.ac.uk/biotic/ . Accessed 2012 July 28

[66] MarLIN (2009) arine Life Information Network. Plymouth: Marine Biological Association of the United Kingdom. Available: http://www.marlin.ac.uk. Accessed 2012 July 28.

[67] Todd JA (2001) Molluscan Life Habits Database. In: University of Iowa, editor; Neogene Marine Biota of Tropical America. Available: http://eusmilia.geology.uiowa.edu/database/mollusc/mollusclifestyles.htm . Accessed 2012 July 28

[68] AustralianMuseum (2009). Crustacea.Net. Available: http://www.crustacea.net. Accessed 2012 July 28.

[69] Clarke KR, Gorley RN (2006) PRIMER v6: User manual/tutorial. Plymouth, UK: PRIMER-E Ltd. p. 192.

[70] Anderson MJ, Gorley RN, Clarke KR (2008) PERMANOVA+ for PRIMER: guide to software and statistical methods. Plymouth, UK: PRIMER-E Ltd.

[71] McArdle BH, Anderson MJ (2001) Fitting multivariate models to community data: a comment on distance-based redundancy analysis. Ecology 82: 290-297. doi:10.1890/0012-9658(2001)082[0290:FMMTCD]2.0.CO;2.

[72] Farias L, Graco M, Ulloa O (2004) Temporal variability of nitrogen cycling in continental-shelf sediments of the upwelling ecosystem off central Chile. Deep Sea Res II Topical Stud Oceanogr 51: 2491-2505. doi:10.1016/j.dsr2.2004.07.029.

[73] Davenport ES, Shull DH, Devol AH (2012) Roles of sorption and tube-dwelling benthos in the cycling of phosphorus in Bering Sea sediments. Deep Sea Res II Topical Stud Oceanogr: 65–70: 163-172

[74] Rysgaard S, Glud RN, Risgaard-Petersen N, Dalsgaard T (2004) Denitrification and anammox activity in Arctic marine sediments. Limnol Oceanogr 49: 1493-1502. doi:10.4319/lo.2004.49.5.1493.

[75] Thouzeau G, Grall J, Clavier J, Chauvaud L, Jean F et al. (2007) Spatial and temporal variability of benthic biogeochemical fluxes associated with macrophytic and macrofaunal distributions in the Thau lagoon (France). Estuarine Coast Shelf Sci 72: 432-446. doi:10.1016/j.ecss.2006.11.028.

[76] Smith KL Jr., Kaufmann RS, Baldwin RJ, Carlucci AF (2001) Pelagic-Benthic Coupling in the Abyssal Eastern North Pacific: An 8-Year Time-Series Study of Food Supply and Demand. Limnol Oceanogr 46: 543-556. doi:10.4319/lo.2001.46.3.0543.

[77] Lepore K, Moran SB, Grebmeier JM, Cooper LW, Lalande C et al. (2007) Seasonal and interannual changes in particulate organic carbon export and deposition in the Chukchi Sea. J Geophys Res Oceans 112: C10024. doi:10.1029/2006JC003555.

[78] Renaud PE, Morata N, Ambrose WG, Bowie JJ, Chiuchiolo A (2007) Carbon cycling by seafloor communities on the eastern Beaufort Sea shelf. J Exp Mar Biol Ecol 349: 248-260. doi:10.1016/j.jembe.2007.05.021.

[79] Sun MY, Carroll ML, Ambrose WG, Clough LM, Zou L et al. (2007) Rapid consumption of phytoplankton and ice algae by Arctic soft-sediment benthic communities: Evidence using natural and C-13-labeled food materials. J Mar Res 65: 561-588. doi:10.1357/002224007782689094.

[80] Morata N, Renaud PE (2008) Sedimentary pigments in the western Barents Sea: A reflection of pelagic-benthic coupling? Deep Sea Res II Topical Stud Oceanogr 55: 2381-2389. doi:10.1016/j.dsr2.2008.05.004.

[81] Sallon A, Michel C, Gosselin M (2011) Summertime primary production and carbon export in the southeastern Beaufort Sea during the low ice year of 2008. Polar Biol 34: 1989-2005. doi:10.1007/s00300-011-1055-5.

[82] Juul-Pedersen T, Michel C, Gosselin M (2010) Sinking export of particulate organic material from the euphotic zone in the eastern Beaufort Sea. Mar Ecol Prog S 410: 55-70. doi:10.3354/meps08608.

[83] Forest A, Babin M, Stemmann L, Picheral M, Sampei M et al. (2013) Ecosystem function and particle flux dynamics across the Mackenzie Shelf (Beaufort Sea, Arctic Ocean): an integrative analysis of spatial variability and biophysical forcings. Biogeosciences 10: 2833-2866. doi:10.5194/bg-10-2833-2013.

[84] Forest A, Bélanger S, Sampei M, Sasaki H, Lalande C et al. (2010) Three-year assessment of particulate organic carbon fluxes in Amundsen Gulf (Beaufort Sea): Satellite observations and sediment trap measurements. Deep Sea Res I Oceanogr Res Pap 57: 125-142. doi:10.1016/j.dsr.2009.10.002.

[85] Rex MA, Etter RJ, Morris JS, Crouse J, McClain CR et al. (2006) Global bathymetric patterns of standing stock and body size in the deep-sea benthos. Mar Ecol Prog S 317: 1-8. doi:10.3354/meps317001.

[86] Witte U, Aberle N, Sand M, Wenzhöfer F (2003) Rapid response of a deep-sea benthic community to POM enrichment: an in situ experimental study. Mar Ecol Prog S 251: 27-36. doi:10.3354/meps251027.

[87] Archambault P, Snelgrove PVR, Fisher JAD, Gagnon J-M, Garbary DJ et al. (2010) From Sea to Sea: Canada’s Three Oceans of Biodiversity. PLOS ONE 5: e12182. doi:10.1371/journal.pone.0012182. PubMed: 20824204. 20824204 10.1371/journal.pone.0012182 PMC2930843

[88] Smith CR, De Leo FC, Bernardino AF, Sweetman AK, Arbizu PM (2008) Abyssal food limitation, ecosystem structure and climate change. Trends Ecol Evol 23: 518-528. doi:10.1016/j.tree.2008.05.002. PubMed: 18584909. 18584909 10.1016/j.tree.2008.05.002

[89] Hoste E, Vanhove S, Schewe I, Soltwedel T, Vanreusel A (2007) Spatial and temporal variations in deep-sea meiofauna assemblages in the Marginal Ice Zone of the Arctic Ocean. Deep Sea Res I Oceanogr Res Pap 54: 109-129. doi:10.1016/j.dsr.2006.09.007.

[90] Cumming GS, Child MF (2009) Contrasting spatial patterns of taxonomic and functional richness offer insights into potential loss of ecosystem services. Philos Trans R Soc Lond B Biol Sci 364: 1683-1692. doi:10.1098/rstb.2008.0317. PubMed: 19451119. 19451119 10.1098/rstb.2008.0317 PMC2685431

[91] Hooper DU, Chapin FS, Ewel JJ, Hector A, Inchausti P et al. (2005) Effect of biodiversity on ecosystem functioning: A consensus of current knowledge. Ecol Monogr 75: 3-35. doi:10.1890/04-0922.

[92] Villéger S, Miranda JR, Hernandez DF, Mouillot D (2012) Low Functional β-Diversity Despite High Taxonomic β-Diversity among Tropical Estuarine Fish Communities. PLOS ONE 7: e40679. doi:10.1371/journal.pone.0040679. PubMed: 22792395. 22792395 10.1371/journal.pone.0040679 PMC3392234

[93] Cochrane SKJ, Pearson TH, Greenacre M, Costelloe J, Ellingsen IH et al. (2012) Benthic fauna and functional traits along a Polar Front transect in the Barents Sea - Advancing tools for ecosystem-scale assessments. J Mar Syst 94: 204-217. doi:10.1016/j.jmarsys.2011.12.001.

[94] Graf G (1992) Benthic-Pelagic Coupling - A Benthic View. Oceanography Mar Biol 30: 149-190.

[95] Jorgensen BB, Glud RN, Holby O (2005) Oxygen distribution and bioirrigation in Arctic fjord sediments (Svalbard, Barents Sea). Mar Ecol Prog S 292: 85-95. doi:10.3354/meps292085.

[96] Na T, Gribsholt B, Galaktionov OS, Lee T, Meysman FJR (2008) Influence of advective bio-irrigation on carbon and nitrogen cycling in sandy sediments. J Mar Res 66: 691-722. doi:10.1357/002224008787536826.

[97] Witman JD, Cusson M, Archambault P, Pershing AJ, Mieszkowska N (2008) The relation between productivity and species diversity in temperate Arctic marine ecosystems. Ecology 89: S66-S80. doi:10.1890/07-1201.1. PubMed: 19097485. 19097485 10.1890/07-1201.1

[98] Gamfeldt L, Hillebrand H, Jonsson PR (2008) Multiple functions increase the importance of biodiversity for overall ecosystem functioning. Ecology 89: 1223-1231. doi:10.1890/06-2091.1. PubMed: 18543617. 18543617 10.1890/06-2091.1

[99] Braeckman U, Provoost P, Gribsholt B, Van Gansbeke D, Middelburg JJ et al. (2010) Role of macrofauna functional traits and density in biogeochemical fluxes and bioturbation. Mar Ecol Prog S 399: 173-186. doi:10.3354/meps08336.

[100] Nascimento FJA, Naeslund J, Elmgren R (2012) Meiofauna enhances organic matter mineralization in soft sediment ecosystems. Limnol Oceanogr 57: 338-346.

[101] Veit-Köhler G, Guilini K, Peeken I, Sachs O, Sauter EJ et al. (2011) Antarctic deep-sea meiofauna and bacteria react to the deposition of particulate organic matter after a phytoplankton bloom. Deep Sea Res II Topical Stud Oceanogr 58: 1983-1995. doi:10.1016/j.dsr2.2011.05.008.

[102] Jørgensen B (2006) Bacteria and Marine Biogeochemistry. In: Schulz HD Zabel M Marine Geochemistry. Springer: Berlin Heidelberg pp. 169-206.

[103] Boetius A, Damm E (1998) Benthic oxygen uptake, hydrolytic potentials and microbial biomass at the Arctic continental slope. Deep Sea Res I Oceanogr Res Pap 45: 239-275. doi:10.1016/S0967-0637(97)00052-6.

[104] Hunter WR, Veuger B, Witte U (2012) Macrofauna regulate heterotrophic bacterial carbon and nitrogen incorporation in low-oxygen sediments. ISME J 6: 2140-2151. doi:10.1038/ismej.2012.44. PubMed: 22592818. 22592818 10.1038/ismej.2012.44 PMC3475371

[105] Michaud E, Desrosiers G, Aller RC, Mermillod-Blondin F, Sundby B et al. (2009) Spatial interactions in the Macoma balthica community control biogeochemical fluxes at the sediment-water interface and microbial abundances. J Mar Res 67: 43-70. doi:10.1357/002224009788597926.

