# Supplementary material for: Are Hotspots Always Hotspots? The Relationship between Diversity, Resource and Ecosystem Functions in the Arctic
Source: PLoS One. 2013 Sep 10;8(9):e74077. doi: 10.1371/journal.pone.0074077 (PMC3769377; doi:10.1371/journal.pone.0074077)
Supplement: Table S1 — Taxa list. The table presents all taxa identified during this study and the accorded functional traits. (PDF) [file pone.0074077.s001.pdf]

Are hotspots always hotspots? The relationship between diversity, resource and ecosystem functions in the Arctic

Heike Link, Dieter Piepenburg, Philippe Archambault

Table S1. Taxa list. The table presents all taxa identified during this study and the accorded functional traits. For details on functional trait categories and levels, see Table 2.

**Class**	**Species/taxon**	Feeding	Size	Motility	Bioturbation
Oligochaeta	*Oligochaeta sp.*	S	L	M	T
Polychaeta	*Ophryotrocha sp.*	O	S	M	S
Polychaeta	*Schistomeringos caeca*	C	S	M	B
Polychaeta	*Schistomeringos rudolphii*	C	M	M	B
Polychaeta	*Lumbrineris scopa*	OC	L	M	G
Polychaeta	*Lumbrineris sp.*	OC	M	M	G
Polychaeta	*Lumbrineris sp. 1*	OC	M	M	G
Polychaeta	*Lumbrineris sp. 2*	OC	M	M	G
Polychaeta	*Lumbrineris sp. 3 cf. fauchaldi*	OC	L	M	G
Polychaeta	*Lumbrineris sp. 4 cf. fragilis*	OC	L	M	G
Polychaeta	*Lumbrineris tetraura/impatiens*	C	L	M	G
Polychaeta	*Nothria conchylega*	O	L	H	S
Polychaeta	*Paradiopatra quadricuspis*	OC	L	H	S
Polychaeta	*Chrysopetalidae sp.*	C	M	M	S
Polychaeta	*Hesionidae sp.*	O	M	M	B
Polychaeta	*Nereimyra sp.*	C	M	M	B
Polychaeta	*Aglaophamus malmgreni*	C	M	M	G
Polychaeta	*Bipalponephtys neotena*	C	M	M	G
Polychaeta	*Micronephtys minuta*	C	S	M	G
Polychaeta	*Nephtyidae sp. 2*	C	M	M	G
Polychaeta	*Nephtyidae sp. 1 cf. Nephtys*	C	M	M	G
Polychaeta	*Nephtys ciliata*	C	L	M	G
Polychaeta	*Pholoe longa*	C	M	M	B
Polychaeta	*Pholoe sp.*	C	S	M	B
Polychaeta	*Eteone flava/longa*	CO	L	M	B
Polychaeta	*Eteone sp.*	CO	L	M	B
Polychaeta	*Phyllodoce sp.*	CO	L	M	B
Polychaeta	*Bylgides sarsi*	C	L	M	S
Polychaeta	*Eucranta sp.*	C	L	M	S
Polychaeta	*Eucranta villosa*	C	L	M	S
Polychaeta	*Gattyana cirrosa*	C	L	M	S
Polychaeta	*Polynoidae*	C	L	M	B
Polychaeta	*Polynoidae sp. 1*	C	L	M	B
Polychaeta	*Clavodorum sp.*	D	S	M	S
Polychaeta	*Sphaerodoropsis sp. 2*	D	S	M	S
Polychaeta	*Sphaerodoropsis sp. 1 cf. furca*	D	S	M	S
Polychaeta	*Sphaerodorum gracilis*	D	S	M	S
Polychaeta	*Anguillosyllis pupa*	D	S	M	S
Polychaeta	*Anguillosyllis sp.*	D	M	M	S
Polychaeta	*Erinaceusyllis sp.*	D	S	M	S
Polychaeta	*Exogone naidina*	D	S	M	S
Polychaeta	*Exogoninae sp.*	D	S	M	S
Polychaeta	*Prosphaerosyllis sp. nov.*	D	S	M	S
Polychaeta	*Streptosyllis sp. nov. 1*	CO	S	M	S
Polychaeta	*Streptosyllis sp. nov. 2*	CO	S	M	S
Polychaeta	*Syllis sp.*	C	M	M	S
Polychaeta	*Galathowenia*	D	M	H	T
Polychaeta	*Galathowenia oculata*	D	M	H	T
Polychaeta	*Myriochele heeri*	D	M	H	T

Table S1 continued					
**Class**	**Species/taxon**	Feeding	Size	Motility	Bioturbation
Polychaeta	*Myriochele olgae*	D	M	H	T
Polychaeta	*Myrioglobula malmgreni*	DF	L	H	T
Polychaeta	*Owenia borealis*	DF	L	H	T
Polychaeta	*Owenia polaris*	DF	L	H	T
Polychaeta	*Owenia sp.*	DF	L	H	T
Polychaeta	*Chone sp.*	F	L	S	T
Polychaeta	*Euchone analis*	F	L	S	T
Polychaeta	*Euchone incolor*	F	L	S	T
Polychaeta	*Jasmineira schaudinni*	F	L	S	T
Polychaeta	*Jasmineira sp.*	F	L	S	T
Polychaeta	*Oriopsis sp.*	F	L	S	T
Polychaeta	*Sabellidae sp.*	F	L	S	T
Polychaeta	*Spirorbis sp.*	F	M	S	S
Polychaeta	*Capitella capitata*	S	L	H	G
Polychaeta	*Capitellidae sp.*	S	L	H	G
Polychaeta	*Hetero- or Mediomastus*	S	L	H	G
Polychaeta	*Heteromastus filiformis*	S	L	H	G
Polychaeta	*Heteromastus sp.*	S	L	H	G
Polychaeta	*Notomastus sp.*	S	L	M	B
Polychaeta	*Cossura pygodactyla*	S	M	H	T
Polychaeta	*Cossura sp.*	S	M	M	B
Polychaeta	*Asychis biceps*	S	L	S	T
Polychaeta	*Asychis sp. 1*	S	L	S	T
Polychaeta	*Clymenura polaris*	S	L	S	T
Polychaeta	*Clymenura sp. 1*	S	L	S	T
Polychaeta	*Lumbriclymeninae sp.*	S	L	S	T
Polychaeta	*Maldane arctica*	S	L	S	T
Polychaeta	*Maldane glebifex*	S	L	S	T
Polychaeta	*Maldane sarsi*	S	L	S	T
Polychaeta	*Maldane sp. (arctica + sarsi)*	S	L	S	T
Polychaeta	*Maldanidae sp. 1*	S	L	S	T
Polychaeta	*Maldanidae sp. 2*	S	L	S	T
Polychaeta	*Maldanidae sp. 3*	S	L	S	T
Polychaeta	*Maldanidae sp. 4*	S	L	S	T
Polychaeta	*Maldanidae sp. 5*	S	L	S	T
Polychaeta	*Maldanidae sp. 6*	S	L	S	T
Polychaeta	*Maldanidae spp.*	S	L	S	T
Polychaeta	*Nicomache cf. lumbricalis*	S	L	S	T
Polychaeta	*Nicomache quadrispinata*	S	L	S	T
Polychaeta	*Nicomachenae/Petaloproctus*	S	L	S	T
Polychaeta	*Petaloproctus tenuis*	S	L	S	T
Polychaeta	*Praxillela affinis*	S	L	S	T
Polychaeta	*Praxillela gracilis*	S	L	S	T
Polychaeta	*Praxillela/axiothella*	S	L	S	T
Polychaeta	*Ophelina cylindricaudata*	S	M	M	G
Polychaeta	*Ophelina sp. 2*	S	M	M	G
Polychaeta	*Ophelina sp. 1 cf. breviata*	S	M	M	G
Polychaeta	*Scoloplos sp. 2*	S	L	M	B
Polychaeta	*Scoloplos sp. 1 cf. acutus ou armiger*	S	L	M	B
Polychaeta	*Aricidea fragilis*	D	M	H	B
Polychaeta	*Aricidea nolani*	D	M	H	G
Polychaeta	*Aricidea quadrilobata*	D	M	H	G
Polychaeta	*Aricidea sp. 1*	D	M	H	G
Polychaeta	*Aricidea sp. 2*	D	M	H	G
Polychaeta	*Aricidea sp. 3 cf. hartmani*	D	M	H	G
Polychaeta	*Aricidea sp. 4*	D	M	H	G
Polychaeta	*Aricidea suecica*	DF	M	H	G
Polychaeta	*Levinsenia (Paraonis) gracilis*	D	M	H	G

Table S1 continued					
**Class**	**Species/taxon**	Feeding	Size	Motility	Bioturbation
Polychaeta	*Paraonidae*	D	M	H	G
Polychaeta	*Scalibregma inflatum*	S	S	M	B
Polychaeta	*Scalibregmatidae sp.*	S	S	M	B
Polychaeta	*Apistobranchus sp. cf. tullbergi*	D	M	H	T
Polychaeta	*Spiochaetopterus sp. cf. typicus*	DF	L	S	T
Polychaeta	*Dipolydora caulleryi*	DF	M	H	T
Polychaeta	*Dipolydora sp.*	DF	M	H	T
Polychaeta	*Laonice sp. cf. cirrata*	DF	M	H	T
Polychaeta	*Polydora/Dipolydora*	DF	M	H	T
Polychaeta	*Prionospio cirrifera*	DF	M	H	T
Polychaeta	*Prionospio sp.*	DF	M	H	T
Polychaeta	*Prionospio steenstrupi*	DF	M	H	T
Polychaeta	*Spio sp.*	DF	M	H	T
Polychaeta	*Spionidae w/o branchia*	D	M	H	T
Polychaeta	*Trochochaeta multisetosa*	D	L	S	T
Polychaeta	*Trochochaeta watsoni*	D	L	S	T
Polychaeta	*Amage gallasii*	DF	M	M	B
Polychaeta	*Amage sp. cf. auricula*	DF	M	H	B
Polychaeta	*Ampharete finmarchica*	D	L	S	T
Polychaeta	*Ampharete sp.*	D	L	S	T
Polychaeta	*Ampharetidae sp. 1*	D	L	S	T
Polychaeta	*Ampharetidae sp. 2 = Amythasides*	D	L	S	T
Polychaeta	*Ampharetidae sp. 3*	D	L	S	T
Polychaeta	*Ampharetidae juv*	D	M	S	T
Polychaeta	*Ampharetinae sp.*	D	L	S	T
Polychaeta	*Ampharetinae sp. 1*	D	L	S	T
Polychaeta	*Ampharetinae sp. 2*	D	L	S	T
Polychaeta	*Ampharetinae sp. 3*	D	L	S	T
Polychaeta	*Ampharetinae sp. 4*	D	L	S	T
Polychaeta	*Amphicteis gunneri*	D	L	S	T
Polychaeta	*Auchenoplax sp.*	D	L	S	T
Polychaeta	*Glyphanostomum pallescens*	D	L	S	T
Polychaeta	*Pterolysippe vanelli*	D	M	H	B
Polychaeta	*Sabellides borealis*	DF	L	S	T
Polychaeta	*Aphelochaeta sp.*	D	L	H	B
Polychaeta	*Chaetozone sp. 1 cf. setosa*	D	M	M	B
Polychaeta	*Chaetozone sp. 2*	D	M	M	B
Polychaeta	*Chaetozone/Tharyx*	D	M	M	B
Polychaeta	*Cirratulidae*	D	L	H	B
Polychaeta	*Monticellina sp.*	D	M	H	B
Polychaeta	*Tharyx sp.*	D	M	H	B
Polychaeta	*Brada villosa*	D	L	H	S
Polychaeta	*Diplocirrus hirsutus*	D	M	H	S
Polychaeta	*Diplocirrus sp. 1 cf. longisetosus*	D	M	H	S
Polychaeta	*Flabelligeridae*	D	M	H	S
Polychaeta	*Cistenides hyperborea*	S	L	M	B
Polychaeta	*Paramphitrite sp.*	D	L	S	T
Polychaeta	*Pista sp.*	D	L	S	T
Polychaeta	*Polycirrus arcticus*	D	L	H	T
Polychaeta	*Polycirrus sp.*	D	L	H	T
Polychaeta	*Proclea graffi*	D	L	S	T
Polychaeta	*Proclea malmgreni*	D	L	S	T
Polychaeta	*Terebellidae sp. 1*	D	L	S	T
Polychaeta	*Terebellidae sp. 2*	D	L	S	T
Polychaeta	*Terebellinae (Amphitritinae) sp.*	D	L	S	T
Polychaeta	*Thelepodinae*	D	L	S	T
Polychaeta	*Terebellides bigeniculatus*	D	L	S	T
Polychaeta	*Terebellides cf. williamsae*	D	L	S	T

Table S1 continued					
**Class**	**Species/taxon**	Feeding	Size	Motility	Bioturbation
Polychaeta	*Terebellides sp.*	D	L	S	T
Polychaeta	*Terebellides stroemi*	D	L	S	T
Polychaeta	*Terebellomorpha sp. 1*				
Polychaeta	*Terebellomorpha sp. 2*				
Malacostraca	*Byblis gaimardii*	F	L	H	S
Malacostraca	*Haploops oonah*	F	L	H	S
Malacostraca	*Haploops sp.*	F	L	H	S
Malacostraca	*Haploops tubicola*	F	L	H	S
Malacostraca	*Argissa hamatipes*	S	L	M	B
Malacostraca	*Caprella septentrionalis*	D	L	M	S
Malacostraca	*Gammaridae*	D	M	M	B
Malacostraca	*Paraphoxus oculatus*	D	M	M	B
Malacostraca	*Isaiedae sp.*	F	L	H	B
Malacostraca	*Ischyrocerus megacheir*	F	M	M	B
Malacostraca	*Ischyrocerus sp.*	F	M	M	B
Malacostraca	*Lysianassidae sp.*	O	L	M	S
Malacostraca	*Paracentromedon sp.*	O	L	M	S
Malacostraca	*Paronesimus sp.*	O	L	M	S
Malacostraca	*Aceroides latipes*	C	M	M	B
Malacostraca	*Bathymedon sp. cf. Obtusifrons*	C	M	M	B
Malacostraca	*Oediceropsis brevicornis*	C	M	M	B
Malacostraca	*Oedicerotidae*	C	M	M	B
Malacostraca	*Paroediceros sp.*	C	M	M	B
Malacostraca	*Halice sp.*	S	M	H	B
Malacostraca	*Pardaliscidae sp.*	S	M	H	B
Malacostraca	*Pontoporeia femorata*	D	M	M	B
Malacostraca	*Diastylidae sp. 1*	DF	M	M	S
Malacostraca	*Diastylidae sp. 2*	DF	M	M	S
Malacostraca	*Diastylis lucifera*	DF	M	M	S
Malacostraca	*Diastylis rathkei*	DF	M	M	S
Malacostraca	*Diastylis sp.*	DF	M	M	S
Malacostraca	*Ektonodiastylis nimia/Brachydiastylis*	DF	M	M	S
Malacostraca	*Eudorella emarginata*	DF	M	M	S
Malacostraca	*Eudorella sp.*	DF	M	M	S
Malacostraca	*Eudorellopsis integra*	DF	M	M	S
Malacostraca	*Leucon acutirostris*	DF	M	M	S
Malacostraca	*Leucon fulvus*	DF	M	M	S
Malacostraca	*Leucon nasicus*	DF	M	M	S
Malacostraca	*Leucon sp.*	DF	M	M	S
Malacostraca	*Leuconidae sp. 1*	DF	M	M	S
Malacostraca	*other 1*	DF	M	M	S
Malacostraca	*other 2*	DF	M	M	S
Malacostraca	*Saduria sabini*	C	L	M	S
Malacostraca	*Desmosoma lineare*	S	S	M	B
Malacostraca	*Eugerda tenuimana*	S	S	M	B
Malacostraca	*Caecognathia stygia*	P	M	H	S
Malacostraca	*Gnathia sp.*	P	M	H	S
Malacostraca	*Synidotea bicuspida*	D	L	M	S
Malacostraca	*Ilyarachna hirticeps*	C	M	M	S
Malacostraca	*Pleurogonium spinosissimum*	S	M	M	B
Malacostraca	*Leptognathia sp.*	D	M	H	S
Malacostraca	*Akanthophoreus gracilis*	D	S	H	S
Malacostraca	*Akanthophoreus sp. cf. disa*	D	S	H	S
Malacostraca	*Pseudotanais affinis*	D	M	H	S
Malacostraca	*Pseudotanais forcipatus*	D	M	H	S
Malacostraca	*Pseudotanais sp.*	D	M	H	S
Malacostraca	*Pseudosphyrapus serratus*	C	M	M	B

Table S1 continued					
**Class**	**Species/taxon**	Feeding	Size	Motility	Bioturbation
Malacostraca	*Typhlotanais sp.*	D	S	H	S
Malacostraca	*Tanaidae*	D	S	H	S
Ostracoda	*Cypridininae*	CO	S	M	S
Ostracoda	*Philomedes sp. 1*	D	S	M	S
Ostracoda	*Philomedes sp. 2 cf. brenda*	D	S	M	S
Ostracoda	*Scleroconcha sp.*	D	S	M	S
Ostracoda	*Myodocopida*	D	S	M	S
Ostracoda	*Hemicytheridea sp.*	CO	S	M	S
Ostracoda	*Sarsicytheridea sp.*	CO	S	M	S
Ostracoda	*Trachyleberididea sp.*	CO	S	M	S
Ostracoda	*Acanthocythereis sp. cf. dunelmensis*	CO	S	M	S
Ostracoda	*Podocopida (Ostracoda B)*	CO	S	M	S
Pycnogonida	*Nymphon sp. 1 cf. hirtum*	C	L	M	S
Pycnogonida	*Nymphon sp. 2 cf hirtipes*	C	L	M	S
Pycnogonida	*Nymphon sp. 3*	C	L	M	S
Bryozoa	*Bryozoa*	F	L	S	S
Gymnolemata	*Notoplites sp.*	F	M	S	S
Gymnolemata	*Eucratea loricata*	F	L	S	S
Gymnolemata	*Romancheinidae sp.*	F	L	S	S
Gymnolemata	*Alcyonidium sp.*	F	M	S	S
Gymnolemata	*Alcyonidium sp. 2*	F	M	S	S
Priapulida	*Priapulida*	S	L	H	B
Priapulida	*Priapulopsis bicaudatus*	S	L	H	B
Ascidiacea	*Phlebobranchia sp.*	F	L	S	S
Ascidiacea	*Polycarpa*	F	M	S	S
Ascidiacea	*Ascideacea sp. 1*	F	M	S	S
Ascidiacea	*Ascideacea sp. 2*	F	M	S	S
Anthozoa	*Actinostolidae sp.*	FCO	L	S	S
Anthozoa	*Edwardsidae*	C	M	H	B
Anthozoa	*Athenaria sp. cf. halcampa*	C	M	H	B
Anthozoa	*Actinaria sp.*	C	M	H	S
Anthozoa	*Cerianthidae sp.*	C	L	H	B
Anthozoa	*Anthozoa sp. 1*	C	M	H	S
Anthozoa	*Anthozoa sp. 2*	C	M	H	S
Hydrozoa	*Anthoathecata*	FCO	L	S	S
Hydrozoa	*Obelia sp.*	FCO	L	S	S
Hydrozoa	*Sertularia sp.*	FCO	L	S	S
Hydrozoa	*Sertulariidae spp.*	FCO	L	S	S
Hydrozoa	*Thuiaria sp.*	FCO	L	S	S
Hydrozoa	*Tiaropsis multicirrata*	FCO	M	M	S
Hydrozoa	*rhizome*	FCO	L	S	S
Asteroidea	*Asteroidea*	C	L	M	S
Asteroidea	*Ctenodiscus crispatus*	DO	L	M	B
Echinodermata	*Echinoderm larvae*	D	L	M	S
Holothuroidea	*Holothuridae sp. 1*	D	M	M	B
Holothuroidea	*Holothuridaesp. 2*	D	M	M	B
Holothuroidea	*Holothuridae sp. 3*	D	M	M	B
Ophiuroidea	*Ophiocten sericeum*	DO	L	M	S
Ophiuroidea	*Ophiuroidea*	DO	L	M	S
Enteropneusta	*Enteropneusta*	D	L	M	B
Bivalvia	*Cuspidaria sp. cf. glacialis*	C	L	H	B
Bivalvia	*Cuspidariidae*	C	M	H	B
Bivalvia	*Bathyarca sp.*	F	M	S	S
Bivalvia	*Astarte sp. 1 cf. elliptica ou esquimati*	F	L	H	B
Bivalvia	*Astarte sp. 2 cf. montagui*	F	L	H	B
Bivalvia	*Limidae*	F	M	S	S
Bivalvia	*Bivalvia sp. 2 (Thyasiridae)*	F	M	H	B

Table S1 continued					
**Class**	**Species/taxon**	Feeding	Size	Motility	Bioturbation
Bivalvia	*Thyasira sp. (east)*	F	M	H	B
Bivalvia	*Thyasira sp. (west)*	F	M	H	B
Bivalvia	*Crenella faba*	F	L	S	S
Bivalvia	*Dacrydium*	F	M	S	S
Bivalvia	*Dacrydium viviparum*	F	M	S	S
Bivalvia	*Musculus glacialis*	F	L	S	S
Bivalvia	*Musculus niger*	F	L	S	S
Bivalvia	*Mytilidae*	F	M	S	S
Bivalvia	*Portlandia sp. cf. frigida*	S	M	H	B
Bivalvia	*Ennucula tenuis*	S	M	M	B
Bivalvia	*Nucula sp.*	S	M	M	B
Bivalvia	*Montacuta sp.*	F	M	S	B
Bivalvia	*Montacutidae sp.*	F	M	S	B
Bivalvia	*Macoma calcarea*	DF	L	H	B
Bivalvia	*Bivalvia decalcifié*	DF	M	H	B
Bivalvia	*Bivalvia sp. 1*	DF	M	H	B
Bivalvia	*Bivalvia sp. 3*	DF	M	H	B
Caudofoveata	*Chaetoderma*	CO	L	M	B
Gastropoda	*Tachyrhynchus erosus*	F	L	M	S
Gastropoda	*Cylichna sp. cf. alba*	C	M	M	S
Gastropoda	*Philinoidea*	C	M	M	S
Gastropoda	*Retusa obtusa*	C	M	M	S
Gastropoda	*Heterobranchia*	C	M	M	S
Gastropoda	*Pyramidelloidea*	O	M	M	S
Gastropoda	*Buccinidae*	C	L	M	S
Gastropoda	*Gastropoda*	D	M	M	S
Scaphopoda	*Scaphopoda*	C	M	H	B
Nematoda	*Nematodes*	S	S	M	G
Nemertea	*Nemerti*	C	L	M	S
Demospongiae	*Demospongiae*	F	M	S	S
Sipuncula	*Sipunculida*	D	M	M	T
	Foraminifera	D	S	S	S
