# Supplementary material for: Are Hotspots Always Hotspots? The Relationship between Diversity, Resource and Ecosystem Functions in the Arctic
Source: PLoS One. 2013 Sep 10;8(9):e74077. doi: 10.1371/journal.pone.0074077 (PMC3769377; doi:10.1371/journal.pone.0074077)
Supplement: Table S2 — Sediment pigment concentrations (Chl a and phaeopigments ‘Phaeo’), community descriptors (taxonomic richness STax, total abundance N, functional group richness SFunc and Shannon-Wiener Index H'Func) and abiotic variables used in the study. (PDF) [file pone.0074077.s002.pdf]

Are hotspots always hotspots? The relationship between diversity, resource and ecosystem functions in the Arctic

Heike Link, Dieter Piepenburg, Philippe Archambault

Table S2. Sediment pigment concentrations (Chl *a* and phaeopigments 'Phaeo'), community descriptors (taxonomic richness S_{Tax}, total abundance N, functional group richness S_{Func} and Shannon-Wiener Index H'_{Func}) and abiotic variables used in the study.

Regime	Site	Year	Chl *a* [µg g^{-1}]	Phaeo [µg g^{-1}]	S_{Tax}	N [ind.]	S_{Func}	H'_{Func}	Depth [m]	Ice-melt [d_{Julian}]
Hotspots	MD-C	2008	23.33	36.44	31	129	23	2.67	45	161
			14.25	31.96	20	172	16	2.25	45	161
			32.44	43.58	32	142	24	2.57	45	161
		2009	3.55	16.93	25	89	19	1.82	47	160
			3.82	9.15	15	75	14	1.84	47	160
			3.89	10.92	19	96	16	2.09	47	160
	AG-CW	2008	0.53	14.66	22	53	16	2.38	206	182
			1.11	15.43	28	98	21	2.11	206	182
			0.87	15.30	24	89	18	1.74	206	182
		2009	1.37	10.81	30	55	19	2.63	154	202
			0.90	10.71	28	87	23	2.67	154	202
			0.79	12.06	23	60	18	2.60	154	202
	LS-W	2008	18.29	37.72	41	186	32	2.59	353	217
			14.02	43.00	34	155	24	2.50	353	217
			10.11	40.82	45	197	31	2.62	353	217
		2009	6.92	33.98	42	253	29	1.95	331	146
			3.57	18.04	36	262	25	1.93	331	146
			4.55	23.68	33	274	23	1.78	331	146
	LS-E	2008	3.21	26.74	36	253	25	1.90	707	182
			4.19	33.80	41	361	25	1.77	707	182
			3.33	31.34	29	238	21	1.50	707	182
		2009	2.73	32.27	31	308	20	1.51	786	167
			1.50	21.65	26	485	18	0.85	786	167
			1.40	19.26	28	457	20	0.95	786	167
	NW-C	2008	3.53	35.34	27	978	19	0.65	444	147
			1.77	12.42	26	996	19	0.77	444	147
			1.88	11.78	18	741	13	0.72	444	147
		2009	3.21	21.71	41	380	25	2.08	451	153
			2.86	22.85	38	566	25	1.51	451	153
			2.98	25.06	40	457	29	1.90	451	153
	NW-E	2008	2.04	14.78	38	356	27	1.33	668	147
			2.51	20.78	33	355	24	1.15	668	147
			2.56	19.37	37	358	27	1.57	668	147
		2009	1.43	25.64	25	434	17	0.93	669	177
			1.08	19.24	24	339	20	1.18	669	177
			0.68	15.57	28	359	22	1.07	669	177
Coldspots	MS-C	2008	0.43	7.10	28	94	22	2.00	318	175
			0.38	6.98	27	74	19	2.23	318	175
			0.50	6.41	16	78	15	1.35	318	175
		2009	0.04	1.27	10	427	9	0.24	577	216
			0.08	2.24	13	449	12	0.27	577	216
			0.04	1.87	10	407	8	0.18	577	216
	AG-CC	2008	0.09	6.84	9	220	9	0.29	596	161
			0.14	6.77	12	173	10	0.47	596	161
			0.22	7.65	15	244	12	0.52	596	161
		2009	0.17	3.31	9	174	9	0.36	559	195
			0.21	3.48	7	212	6	0.20	559	195
			0.21	4.24	8	204	8	0.22	559	195
	BB-N	2008	0.18	3.63	18	409	15	0.40	795	203
			0.06	4.28	16	403	15	0.40	795	203
			0.14	3.78	16	463	14	0.37	795	203
		2009	0.93	4.54	17	402	16	0.52	810	160
			0.42	3.19	18	274	15	0.52	810	160
			0.39	2.00	15	242	12	0.59	810	160
